# ﻿Caribbean Amphipoda (Crustacea) of Panama. Part V: parvorder Amphilochidira

**DOI:** 10.3897/zookeys.1259.165130

**Published:** 2025-11-10

**Authors:** Abigail R. Dugger, Kristine N. White

**Affiliations:** 1 Georgia College & State University, Department of Biological and Environmental Sciences, Aquatic Sciences Center, Milledgeville, GA 31061 USA Georgia College & State University Milledgeville United States of America

**Keywords:** Amphilochidae, Amphilochidira, Bocas del Toro, identification key, Leucothoidae, new species, Sebidae, Stenothoidae

## Abstract

The parvorder Amphilochidira includes 1,152 described species in 24 families. Amphilochidiran amphipods retain many plesiomorphic character states and are thought to represent the ancestral state of amphipods. Many amphipods in this parvorder cling to substrates such as algae or sponges. Nineteen species from four families within the parvorder are documented from Bocas del Toro, Panama, including one new species, *Apolochus dragensis***sp. nov.** Range extensions are documented for eight species, including two species previously documented only from the Pacific Ocean. All species are diagnosed and an identification key to the Amphilochidira amphipods of Panama is provided herein.

## Abstract (es)

El parvorden Amphilochidira incluye 1,152 especies descritas en 24 familias. Los anfípodos Amphilochidira conservan muchos estados plesiomórficos y se cree que representan el estado ancestral de los anfípodos. Muchos anfípodos de este parvorden se adhieren a sustratos como algas o esponjas. Se documentan diecinueve especies de cuatro familias dentro del parvorden en Bocas del Toro, Panamá, incluyendo una nueva especie, *Apolochus dragensis***sp. nov.** Se registran extensiones de distribución para ocho especies, incluyendo dos previamente documentadas solo en el Océano Pacífico. Se diagnostican todas las especies y se proporciona una clave de identificación para los anfípodos Amphilochidira de Panamá.

## ﻿Introduction

Amphilochidira Boeck, 1871 is a parvorder consisting of 1,152 species distributed worldwide (Horton et al. 2025). A synapomorphy shared by most families in the Amphilochidira is having the pereopod 4 carpus shorter than the propodus. Amphipods in the parvorder also share the following characteristics: sparsely setose antenna 2; gnathopod 2 simple, subchelate (chelate in Didymocheliidae Bellan-Santini & Ledoyer, 1987); pereopods 6 and 7 not extremely elongate; urosomites 1–3 separate (fused in Sebidae Walker, 1907), and an entire telson (Lowry and Myers 2017). Amphipods in this parvorder have retained many plesiomorphic character states, suggesting that this parvorder represents the ancestral state of amphipods, many of whom cling to substrates such as algae or sponges (Lowry and Myers 2017).

According to Horton et al. (2025) the Amphilochidira comprises 24 families: Acanthonotozomatidae Stebbing, 1906 (11 spp.); Acanthonotozomellidae Coleman & Barnard, 1991 (8 spp.); Amathillopsidae Pirlot, 1934 (24 spp.); Amphilochidae Boeck, 1871 (93 spp.); Bolttsiidae Barnard & Karaman, 1987 (2 spp.); Cressidae Stebbing, 1899 (10 spp.); Cyproideidae Barnard, 1974 (46 spp.); Didymocheliidae (5 spp.); Dikwidae Coleman & Barnard, 1991 (2 spp.); Epimeriidae Boeck, 1871 (91 spp.); Iphimediidae Boeck, 1871 (105 spp.); Lafystiidae Sars, 1895 (6 spp.); Laphystiopsidae Stebbing, 1899 (8 spp.); Leucothoidae Dana, 1852 (205 spp.); Nihotungidae Barnard, 1972 (3 spp.); Ochlesidae Stebbing, 1910 (22 spp.); Odiidae Coleman & Barnard, 1991 (20 spp.); Pleustidae Buchholz, 1874 (141 spp.); Sebidae (25 spp.); Seborgiidae Holsinger, 1980 (in Holsinger and Longley 1980) (9 spp.); Sicafodiidae Just, 2004 (3 spp.); Stenothoidae Boeck, 1871 (284 spp.); Stilipedidae Holmes, 1908 (27 spp.); and Vicmusiidae Just, 1990 (2 spp.).

Prior to this study, 37 Amphilochidiran species in six families were documented from Caribbean waters: Amphilochidae, Cyproideidae, Leucothoidae, Ochlesidae, Sebidae, and Stenothoidae (LeCroy et al. 2009; Miloslavich et al. 2010; Martín et al. 2013). Of the 37 species, eleven leucothoid species had been previously documented in Caribbean Panama (*Anamixis cavatura* Thomas, 1997; *Anamixis vanga* Thomas, 1997; *Leucothoe ashleyae* Thomas & Klebba, 2006; *Leucothoe barana* Thomas & Klebba, 2007; *Leucothoe flammosa* Thomas & Klebba, 2007; *Leucothoe kensleyi* Thomas & Klebba, 2006; *Leucothoe laurensi* Thomas & Ortiz, 1995; *Leucothoe saron* Thomas & Klebba, 2007; *Leucothoe ubouhu* Thomas & Klebba, 2007; *Leucothoe tunica* White, 2019 (as *Leucothoe* sp. C), and *Leucothoe wuriti* Thomas & Klebba, 2007). *Leucothoe panpulco* Barnard, 1961 was documented from the Pacific side of Panama at a depth of 3570 m (Barnard 1961). Nineteen Amphilochidiran species were collected during this study, including all but two of the leucothoid species previously reported from Panama (*L. panpulco* and *L. saron*).

## ﻿Materials and methods

Coral rubble, sponges, ascidians, algae, sand, seagrass, hydroids, mangrove scrapings, and buoy scrapings were collected at 21 sites around Bocas del Toro, Panama at depths of 0–15 m. Coral rubble samples were elutriated with freshwater and other substrates were hand-picked to remove amphipods. Living amphipods were sorted into morphospecies, placed in clove oil for imaging, and preserved in 99.5% EtOH. Preserved specimens were examined in glycerol after being measured from the tip of the rostrum to the base of the telson. Amphipods were dissected using a stereomicroscope and illustrated using an Olympus BH2 differential interference contrast microscope with an Olympus BH2-DA drawing tube attached. Pencil drawings were digitally inked using a Wacom Intuos Pro Pen tablet following the methods of Coleman (2003) in Adobe Illustrator 2020. Abbreviations used in figures are as follows: **H**, habitus; **Hd**, head; **A**, antenna; **C**, coxa; **G**, gnathopod; **P**, pereopod; **E**, epimeron; **Pl**, pleopod; **U**, uropod; **Ur**, urosome; **a**, anamorph; **l**, leucomorph. Size ranges of each species collected from Bocas del Toro, Panama are provided at the beginning of each material examined section. Specimens are deposited in the Smithsonian Institution, U.S. National Museum of Natural History (**USNM**), and the Gulf Coast Research Laboratory Museum (**GCRL**).

## ﻿Results

### ﻿Taxonomic account

**Parvorder Amphilochidira Boeck, 1871**

**Superfamily Amphilochoidea Boeck, 1871**

**Family Amphilochidae Boeck, 1871**

#### ﻿Genus Apolochus

Taxon classificationAnimaliaAmphipodaAmphilochidae

Hoover & Bousfield, 2001

C34B7F6D-CA4A-5957-BF3B-2FEC2826FD7D

##### Diagnosis.

Antenna 1 short, subequal in length with or slightly longer than peduncle of antenna 2, peduncle segments 1 and 2 broadened. Gnathopods 1 and 2 strongly subchelate. Gnathopod 2 carpus posterior lobe well-developed; propodus palm distinct, palmar angle defined by one or two spines. Pereopods 3 and 4 dactyli shorter than dactyli of pereopods 5–7. Telson subtriangular or linguiform.

#### ﻿ Apolochus dragensis sp. nov.

Taxon classificationAnimaliaAmphipodaAmphilochidae

895C10EA-D724-513C-BE33-3B65B309586F

https://zoobank.org/AC0AD314-A144-4BBE-BF61-C644E550B6D6

Figs 1, 3, 22A

?Apolochus sp. A: LeCroy 2002: 231, fig. 237.
?Amphilochus neapolitanus: McKinney 1978: 137.
?Amphilochus neapolitanus: Thomas 1993: 24, fig. 25.

##### Type locality.

Bocas del Toro, Panama: Drago; 9.4181°N, 82.3375°W; depth 2–3 m; among coral rubble.

##### Distribution.

Panama: Bocas del Toro (present study).

##### Material examined.

***Holotype***: Panama • 1 ♀, 2.3 mm; Bocas del Toro, Drago; 9.4181°N, 82.3375°W; depth 2–3 m; among coral rubble; 9 Aug 2021; K.N. White leg.; USNM 1762913. ***Paratype***: Panama • 1 ♀, 2.4 mm; Bocas del Toro, Swan Caye; 9.4536°N, 82.3000°W; depth 2 m; among coral rubble; 24 June 2023; K.N. White leg.; USNM 1762914.

**Figure 1.**
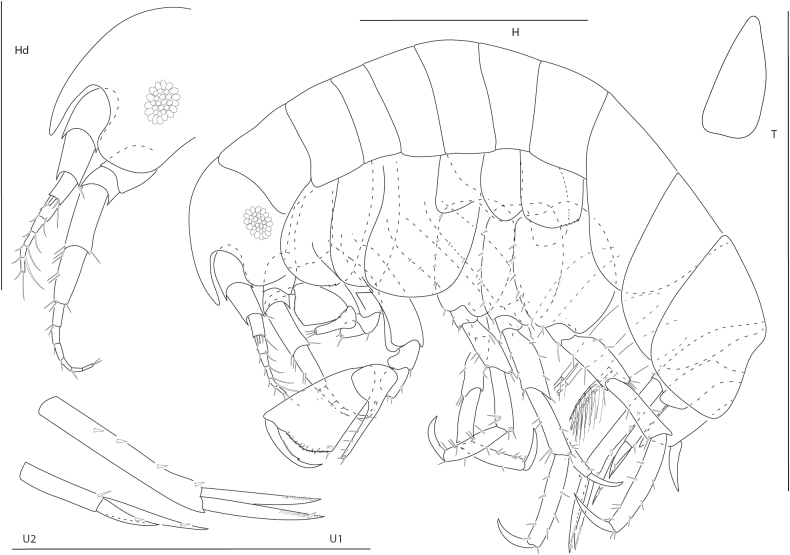
*Apolochus dragensis* sp. nov., female holotype, 2.3 mm, head, habitus, telson, uropod 1, and uropod 2. Scale bars: 0.5 mm.

##### Diagnosis.

Head anteroventral margin evenly rounded. Antenna 1 reaching the end of antenna 2 peduncle, with minute uni-articulate accessory flagellum. Mandibular molar reduced with a single apical spine. Gnathopod 2 carpal lobe nearly reaching palmar angle, propodus lacking anterolateral spines and anterodistal projection, anterodistal corner rounded, palm finely crenulate.

**Figure 2.**
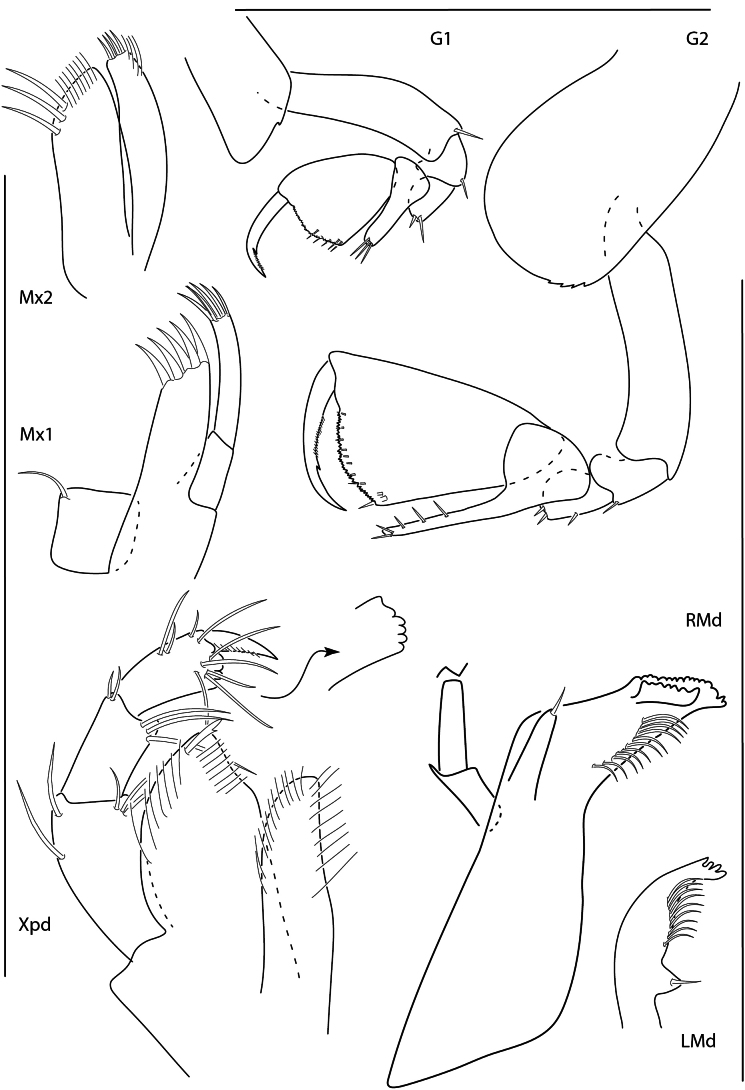
*Apolochus dragensis* sp. nov., female holotype, 2.3 mm, maxilla 2, gnathopod 1 lateral, maxilla 1, gnathopod 2 lateral, maxilliped, and left mandible; female paratype, 2.4 mm, right mandible. Scale bars: 0.5 mm.

##### Description.

**Female** (holotype, 2.3 mm). ***Head*.** Eye medium, oval, darkly pigmented in the center. Head anteroventral margin evenly rounded; rostrum reaching end of antennae 1 peduncle article 1. Antenna 1 shorter than antenna 2, nearly reaching end of antenna 2 peduncle; flagellum 5-articulate; accessory flagellum uni-articulate, minute. Antenna 2 1.3 × length of antenna 1; flagellum 6-articulate. Maxilliped inner plate lined with marginal setae; outer plate broad, margins lined with setae, apical margin with one bifurcate spine-seta, inner margin slightly concave; palp article 3 inner distal margin tuberculate. Maxilla 1 inner plate with 5 marginal spine-setae, palp segment 2 elongate with 5 distal setae. Maxilla 2 plates medium width, both plates lined with thin and medium width setae apically. Mandibular molar reduced, with single apical spine; left mandible with row of nine spine setae; palp missing. Upper and lower lips damaged in dissection.

**Figure 3.**
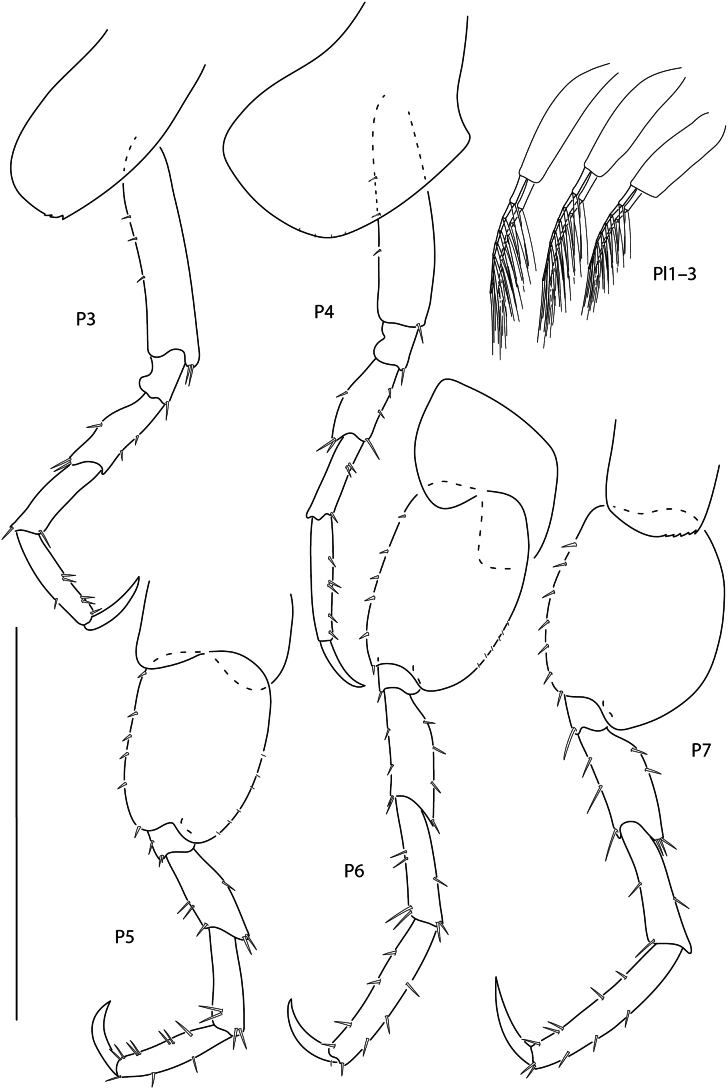
*Apolochus dragensis* sp. nov., female holotype, 2.3 mm, pereopod 3, pereopod 4, pereopod 5, pereopod 6, pereopod 7, and pleopods 1–3. Scale bars: 0.5 mm.

***Pereon*.** Coxae 1 short, ventral margin serrate; coxa 2 rounded, posteroventral margin serrate; coxa 3 anteriorly rounded, posteroventral margin serrate; coxa 4 rounded, posterior margin concave. Gnathopod 1 basis with one posteroventral seta; ischium as wide as long; carpal lobe reaching 0.67 × propodus posterior margin, distal margin with three setae; propodus palm convex, crenulate, with five setae in distal half; dactylus distal inner margin lined with setae. Gnathopod 2, basis bare; carpal lobe reaching palmar angle; propodus lacking anterolateral setae, anterodistal corner rounded, palm convex, crenulate, lined with small setae; dactylus slender, proximal margin lined with setae. Pereopods 3 and 4 dactyli medium, slender. Pereopods 5–7 bases rounded, pereopod 7 basis widest; dactylus medium, slender.

***Pleon*.** Epimera 1–3 rounded, bare. Pleopods, rami longer than peduncle; pleopods 1–3 rami with eight, seven, and six articles, respectively. Uropod 1 slender, peduncle with four marginal spine-setae, 1.6 × length of rami; rami subequal, distal margins lined with setae; inner ramus lacking spine-setae; outer ramus with one spine-seta. Uropod 2 0.5 × length of uropod 1; peduncle with one distal spine-seta, 0.5 × length of inner ramus; inner ramus 1.7 × length of outer ramus, with one distal spine-seta; outer ramus with one distal spine-seta. Uropod 3 missing. Telson subtriangular, length 2.4 × width, apex narrowly rounded.

**Variation** (paratype female, 2.4 mm). Mandibular spine row with eleven spine setae.

**Male** unknown.

##### Etymology.

After the place Drago, Bocas del Toro, Panama, meaning “mouth of the dragon” and referring to the type locality.

##### Ecology and remarks.

This species occurs among coral rubble at 2–3 m depth in Panama. This species is most likely the species called *Amphilochus neapolitanus* (Della Vale, 1893) from the western Atlantic and Caribbean Sea (McKinney 1978; Thomas 1993) and *Apolochus* sp. A (LeCroy 2002) based on the small, non-triturative molar and lack of anterolateral spines on the gnathopod 2 propodus. *Apolochus dragensis* sp. nov. can be differentiated from *Apolochus neapolitanus* based on the drawings from the Mediterranean illustrating a slightly triturative molar with two spines, a more densely setose uropod 2, and a wider telson (Krapp-Schickel 1982). The reduced molar and lack of anterolateral spines on the gnathopod 2 propodus easily distinguish this species from all other species documented from the region who have a triturative molar and anterolateral spines on the gnathopod 2 propodus (*A. pillaii* (Barnard & Thomas, 1983), *A. cassahoya* (McKinney, 1978), and *A. delacaya* (McKinney, 1978)). The new species shares a minute accessory flagellum on antenna 1 with *A. pillaii*, but *A. cassahoya* and *A. delacaya* have an accessory flagellum equal to the length of antenna 1 peduncle article 3. The new species shares a reduced molar with several other species but differs in the following ways: *A. barnardi* and *A. staudei* Hoover & Bousfield, 2001 have short, stout dactyls on pereopods 3 and 4 (vs medium length and slender); *A. borealis* (Enequist, 1949) has a short carpus on gnathopod 2 (vs reaching the palmar angle); and *A. litoralis* (Stout, 1912) has densely spinose uropods (vs few spine-setae). The new species is also easily distinguished from the other *Apolochus* species diagnosed here in having a round head margin (vs acute in A. cf. picadurus (Barnard, 1962) and subquadrate in *A. pillaii*). *Apolochus dragensis* sp. nov. differs from all other described *Apolochus* species in having a reduced molar. Living specimens are translucent with tan coloration on anterior and posterior ends, white opaque coloration on anterior half, rust coloration on pereopods 6 and 7, red stripes on antennae, and red eyes.

#### ﻿ Apolochus cf. picadurus

Taxon classificationAnimaliaAmphipodaAmphilochidae

(Barnard, 1962)

E092FE14-34F3-50F2-81B6-60291BA932DD

Figs 4, 22B

Amphilochus picadurus Barnard, 1962: 126–129, fig. 4.
Apolochus picadurus : Hoover and Bousfield 2001: 15.

##### Material examined.

Panama • 1–2 mm • 3 ♀; Bocas del Toro, Pigeon Key Reef; 9.2693°N, 82.2489°W; depth 0.5–1 m; among *Halimeda* and *Thalassia*; 9 Aug 2005; E. Baiser, T.A. Haney, S. LeCroy leg.; GCRL 6673 • 2 ♀; Bocas del Toro, Crawl Caye; 9.2476°N, 82.1290°W; depth 5–8 m; among coral rubble; 26 June 2023; K.N. White leg.; USNM 1762915.

**Figure 4.**
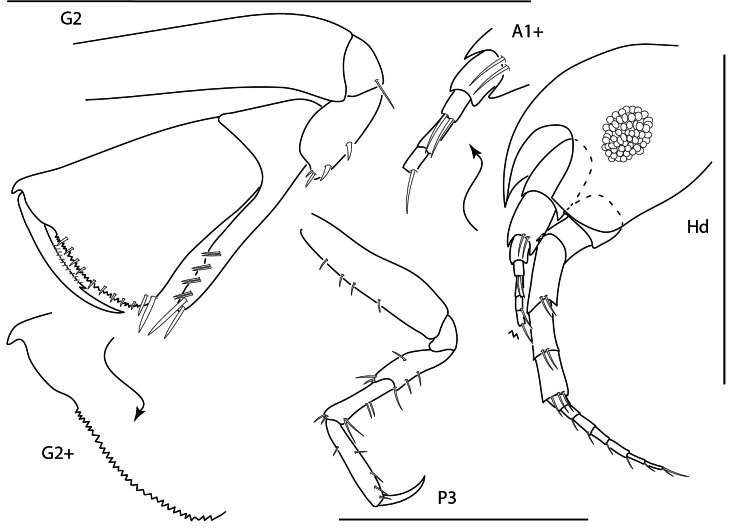
Apolochus cf. picadurus, female, 1.9 mm, gnathopod 2 lateral, pereopod 3, head, and antennae 1. Scale bars: 0.5 mm.

##### Diagnosis.

Head anteroventral margin acute. Antenna 1 shorter than peduncle of antenna 2, lacking accessory flagellum. Gnathopod 2 carpal lobe nearly reaching palmar angle; propodus lacking anterolateral spines, with anterodistal projection. Pereopods 3 and 4 dactyli slender.

##### Distribution.

USA: Southern California (Barnard 1962); Panama: Bocas del Toro (present study).

##### Ecology and remarks.

This species occurs among coral rubble, *Halimeda*, and *Thalassia* at depths of 0.5–8 m in Panama and among green mud and rock in Southern California at depths from 3.6–36 m (Barnard 1962). Panamanian specimens agree closely with the original description. However, the mandibular molar was broken during dissection and cannot be confirmed in Panamanian specimens. The current distribution of this species is from central to Southern California (Barnard 1962). This finding extends the range of this species to include the Caribbean Sea. Analysis of more specimens is needed to confirm the species. Color of living specimens is unknown, ethanol-preserved specimens retain a brown spotted pattern.

#### ﻿ Apolochus pillaii

Taxon classificationAnimaliaAmphipodaAmphilochidae

(Barnard & Thomas, 1983)

EFA867F5-BC21-57A9-A966-B0AC2AAF50BF

Figs 5, 22C

Amphilochus pillaii Barnard & Thomas, 1983: 179–187, figs 1–3.
Apolochus pillaii : Hoover and Bousfield 2001: 15; LeCroy 2002: 230, fig. 239.

##### Material examined.

Panama • 1.3 mm • 1 ♀; Bocas del Toro, Swan Caye; 9.4536°N, 82.3000°W; depth 2 m; among coral rubble; 24 June 2023; K.N. White leg.; USNM 1762916.

**Figure 5.**
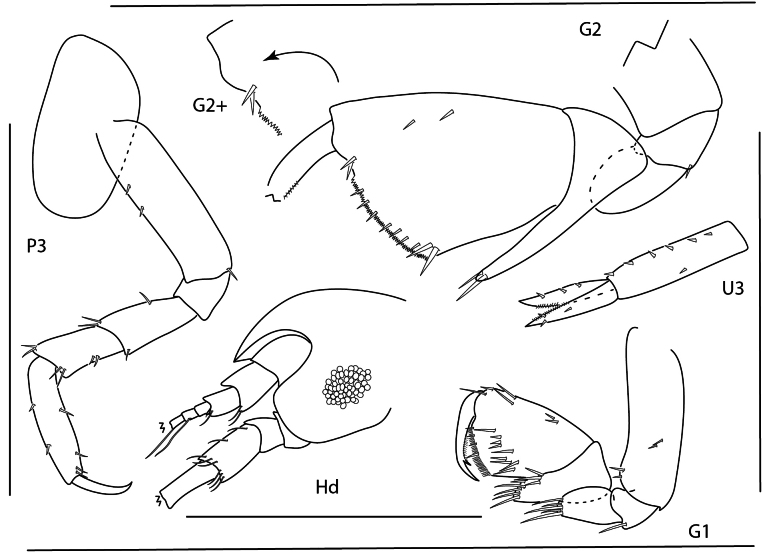
*Apolochus pillaii*, female, 1.3 mm, pereopod 3, gnathopod 2 lateral, head, gnathopod 1 lateral. Scale bars: 0.5 mm.

##### Diagnosis.

Head anteroventral margin subquadrate. Antenna 1 shorter than peduncle of antenna 2 with minute uni-articulate accessory flagellum. Gnathopod 2 carpal lobe clearly not reaching palmar angle; propodus with two anterolateral spines, lacking anterodistal projection, anterodistal margin acute. Pereopods 3 and 4 dactyli slender.

##### Distribution.

USA: Florida Keys (Barnard and Thomas 1983); Belize (Martín et al. 2013); Panama: Bocas del Toro (present study).

##### Ecology and remarks.

This species occurs among coral rubble at a depth of 2 m in Panama and is commensal on *Pterogorgia anceps*, the purple gorgonian in the Florida Keys (Barnard and Thomas 1983). Panamanian specimens agree closely with the original description of this species with the exception of having two anterolateral spines on gnathopod 2 propodus (vs four), which may be due to the smaller size of the Panamanian specimen (1.3 vs 3.3 mm). Living specimens are translucent with tan coloration on anterior and posterior ends, white coloration on pereonites 3 and 4, brown spots on entire body, red stripes on antennae, and red eyes.

### ﻿Family Sebidae Walker, 1907

#### ﻿Genus Seba

Taxon classificationAnimaliaAmphipodaSebidae

Bate, 1863

EF40D07C-5995-56F9-9A14-F4D8E961266A

##### Diagnosis.

Antennae stout, flagella reduced. Eyes absent or poorly developed. Body subcylindrical. Gnathopod 1 chelate or subchelate. Gnathopod 2 chelate. Urosome segments 2 and 3 fused; uropod 3 uniramous. Telson entire.

#### ﻿ Seba cf. tropica

Taxon classificationAnimaliaAmphipodaSebidae

McKinney, 1980

A20BC407-E3ED-54C8-B08D-5BE8C2F9B68E

Figs 6, 22D

Seba A: McKinney 1977: 200–203, pls 47, 48.
Seba tropica McKinney, 1980: 99–102, figs 8, 9; LeCroy 2011: 100, fig. 13.
Caribseba tropica : Shaw 1989: 1885.
Seba n. sp.: Oliva-Rivera and Jiménez-Cueto 1992: 182.

##### Material examined.

Panama • 1.5 mm • 1 juvenile; Bocas del Toro, Crawl Caye; 9.2505°N; 82.1316°W; depth 10 m; among coral rubble and red sponges; 7 Aug 2005; S. DeGrave and M. Salazar leg.; GCRL 6674.

**Figure 6.**
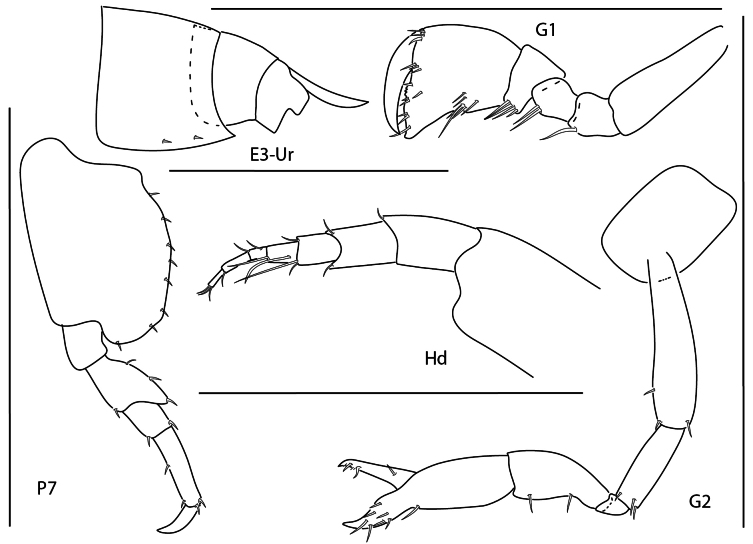
Seba cf. tropica, juvenile, 1.5 mm, epimeron 3 and urosome, gnathopod 1 lateral, pereopod 7, head, gnathopod 2 medial. Scale bars: 0.5 mm.

##### Diagnosis.

Antenna 1 sparsely setose; peduncle article length ratios: 1:1.1:0.4; accessory flagellum lacking. Gnathopod 1 chelate; propodus palm transverse; dactylus proximal margin smooth. Gnathopod 2 article 5 0.7× length of article 6 (measured to distal end). Pereopod 7 basis posterior margin with distoventral lobe. Epimeron 3 posteroventral corner acute, produced.

##### Distribution.

USA: Biscayne Bay, Florida to the Florida Keys (Thomas 1993), Port Isabel, Texas (McKinney 1980); Mexico: Yucatan Peninsula (McKinney 1980; Oliva-Rivera and Jiménez-Cueto 1992 as *Seba* n. sp.); Belize: Carrie Bow Caye (Thomas 1993); Venezuela: Cayo Boca Seca (Martín 2001); Panama: Bocas del Toro (present study).

##### Ecology and remarks.

This species occurs among coral rubble, *Thalassia*, and red sponges at depths to 12 m. Only one small specimen of this species was collected in 2005. This specimen nearly matches the original description of *Seba tropica* but differs in the following characters: antenna 1 setose (vs unarmed) and peduncle article length ratios: 1:1.1:0.4 (vs 1:1.4:0.5). *Seba tropica* is the only described species of *Seba* without an accessory flagellum. This specimen may represent a new species, but this will need to be confirmed with more specimens. Thomas (1993) reports that living specimens are ivory in color.

### ﻿Family Stenothoidae Boeck, 1871

#### ﻿Genus Stenothoe

Taxon classificationAnimaliaAmphipodaStenothoidae

Dana, 1852

8B782364-46D9-5A7B-ABA0-2F5CA049DB67

##### Diagnosis.

Maxilla 1 palp bi-articulate. Gnathopods 1 and 2 subchelate. Coxa 1 reduced; coxa 4 large, shield-like, posteriorly tapered, not covering basis of pereopod 7. Pereopod 5 basis linear. Pereopods 6 and 7 basis expanded. Uropod 3 uniramous, ramus bi-articulate.

#### ﻿ Stenothoe gallensis

Taxon classificationAnimaliaAmphipodaStenothoidae

Walker, 1904

0BC7B530-32E2-5037-A83A-7FD1E7993AEE

Figs 7, 22E

Probolium polyprion : Catta 1876: 15–27, pl. 2, fig. 1 [not Probolium polyprion Costa, 1853].
Stenothoe gallensis Walker, 1904: 261–262, pl. 3, fig. 19; LeCroy 2011: 722, fig. 569; Krapp-Schickel 2015: 8–10.
Stenothoe cattail : Stebbing 1906: 195.
Stenothoe crenulata : Chevreux 1907: 412–413.
Stenothoe valida : Kunkel 1910: 16–18, fig. 5 [not Stenothoe valida Dana, 1853]
Stenothoe sp. A: Rakocinski et al. 1996: 350.

##### Material examined.

Panama • 1–4 mm • 1 ♀; Bocas del Toro, Pandora; 9.3278°N, 82.2222°W; depth 10 m; among coral rubble; 10 Aug 2021; K.N. White leg.; USNM 1762917 • 6 ♂, 37 ♀, 3 juveniles; Bocas del Toro, Hospital Point; 9.3333°N, 82.2185°W; depth 11 m; buoy scraping; 26 June 2023; K.N. White leg.; USNM 1762918 • 3 ♂, 2 ♀; Bocas del Toro, Crawl Caye; depth 0–1 m; buoy scraping; 29 June 2023; K.N. White leg.; USNM 1762919.

**Figure 7.**
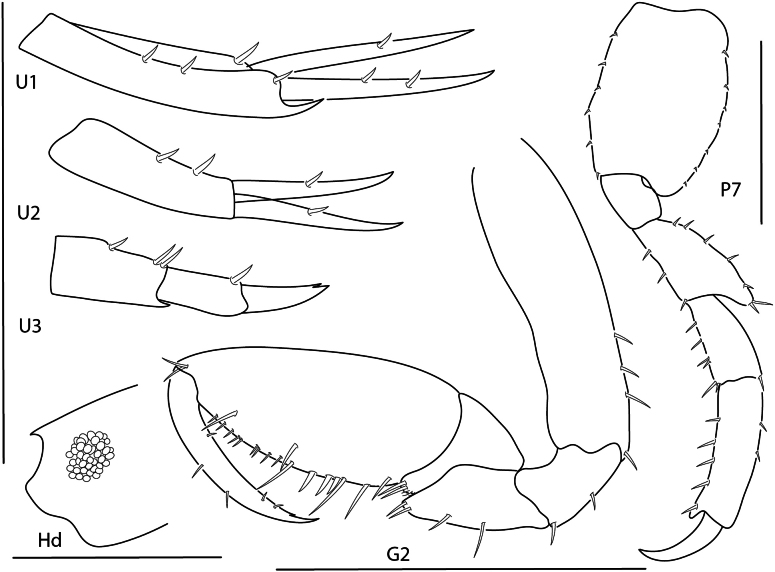
*Stenothoe gallensis*, female, 3 mm, uropods 1–3, head, gnathopod 2 lateral, pereopod 7. Scale bars: 0.5 mm.

##### Diagnosis.

Head anterior margin subtruncate, eye relatively small. Gnathopod 2 of female propodus subovate, palmar angle absent, palm smooth. Gnathopod 2 of male propodus palmar angle absent, with distal palmar tooth (not illustrated). Pereopod 7 merus posterodistal lobe not reaching more than halfway to distal margin of carpus. Uropod 1 peduncle with distoventral spur; inner ramus with one spine-seta. Uropod 3 second article of ramus subequal in length with first article of ramus.

##### Distribution.

This species has been reported worldwide in tropical and warm temperate seas. Western Atlantic distribution: USA: North Carolina (Fox and Bynum 1975), Louisiana (Lewbel et al. 1987), Florida (Culpepper 1969; Thomas 1993; Nelson 1995; Rakocinski et al. 1996; LeCroy 2011), Texas (McKinney 1977); Mexico: Veracruz (McKinney 1977), Quintana Roo (Oliva-Rivera and Jiménez-Cueto 1992); Cuba: Sabana-Camagüey (Ortiz and Lalana 1996); Costa Rica: (Martín et al. 2013); Venezuela: Anzoátegui, Sucre (Martín and Díaz 2003); Puerto Rico (Shoemaker 1935); Virgin Islands (Shoemaker 1935); Panama: Bocas del Toro (present study).

##### Ecology and remarks.

This species occurs among coral rubble and fouling organisms at depths to 11 m. Panamanian specimens agree closely with previous descriptions of *Stenothoe gallensis*. Living specimens are white with mottled mustard coloration, orange antennae, and orange eyes.

#### ﻿ Stenothoe minuta

Taxon classificationAnimaliaAmphipodaStenothoidae

Holmes, 1903

5A68F180-F264-52C3-8F47-B714E1F47B81

Figs 8, 22F

Stenothoe minuta Holmes, 1903: 278; Holmes 1905: 485–486; LeCroy 2011: 724, fig. 572.

##### Material examined.

Panama • 1–2 mm • 1 juvenile; Bocas del Toro, Hospital Point; 9.3336°N, 82.2188°W; depth 15 m; among coral rubble and red algae; 6 Aug 2005; S. DeGrave and M. Salazar leg.; GCRL 6675 • 1 ♀, 1 juvenile; Bocas del Toro, Pandora; 9.3278°N, 82.2222°W; depth 10 m; among coral rubble; 10 Aug 2021; K.N. White leg.; USNM 1762920.

**Figure 8.**
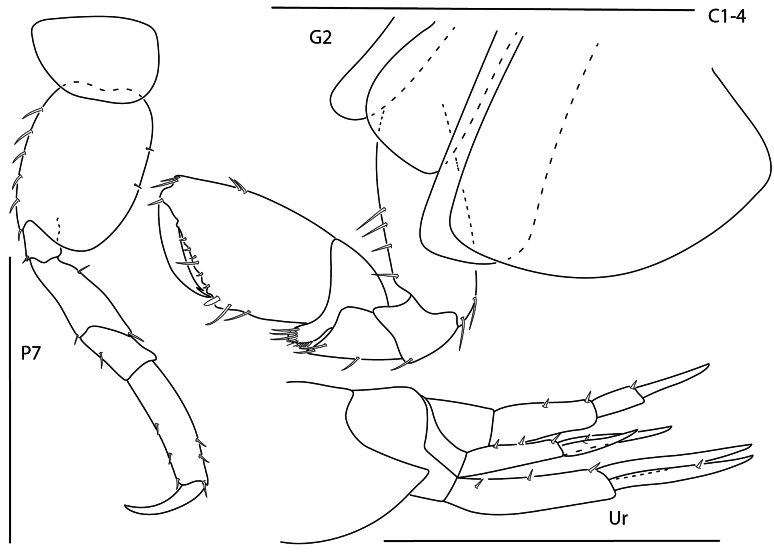
*Stenothoe minuta*, female, 1.5 mm, pereopod 7, gnathopod 2 lateral, coxae 1–4, urosome, uropods 1–3. Scale bars: 0.5 mm.

##### Diagnosis.

Head anterior margin rounded, eye relatively large. Gnathopod 2 of female propodus subrectangular, palmar angle defined by spine, palm crenulate. Gnathopod 2 of male propodus palm and posterior margin distinguishable, lacking distal palmar tooth (not illustrated). Pereopod 7 merus posterodistal lobe not reaching more than halfway to distal margin of carpus. Uropod 1 peduncle lacking distoventral spur; inner ramus bare. Uropod 3 second article of ramus 1.6 × length of first article of ramus.

##### Distribution.

USA: Texas (McKinney 1977); Cape Cod, Massachusetts to northeast Florida (Holmes 1903; Watling and Maurer 1972; Bousfield 1973; Caine 1986), Pine Island Sound, Florida (LeCroy 2011), Apalachee Bay, Florida (Lewis 1984, 1987); Panama: Bocas del Toro (present study).

##### Ecology and remarks.

This species occurs among coral rubble in Panama at depths to 15 m. Previous reports list this species among hydroids, algae, and other fouling organisms (LeCroy 2011). Panamanian specimens agree closely with previous descriptions of this species and can be distinguished by the elongate second article of the uropod 3 ramus. Living specimens are translucent with white spots covering body and red eyes.

#### ﻿ Stenothoe valida

Taxon classificationAnimaliaAmphipodaStenothoidae

Dana, 1853

FF1CA05F-424C-5941-8A12-6D746EC66E7B

Figs 9, 22G

Stenothoe validus Dana, 1853: 924–925, pl. 63, fig. 1.
Probolium polyprion : Costa 1853: 173.
Probolium megacheles : Heller 1866: 13–14, pl. 2, figs 1, 2.
Stenothoe valida : Stebbing 1906: 194; LeCroy 2011: 726, fig. 570; Krapp-Schickel 2015: 40–45, figs 21–23.
Stenothoe assimilis : Chevreux 1908: 4–8, figs 4–6.
Stenothoe ornata : Barnard 1930: 341, fig. 16.

##### Material examined.

Panama • 1–3.5 mm • 1♀; Bocas del Toro, Swan Caye; 9.4533°N, 82.2983°W; depth 3 m; among brown algae, filamentous red algae, and hydroids; 4 Aug 2005; M. Faust, E. Gaiser, T. Haney, S. Richardson, M. Sorenson leg.; GCRL 6676 • 1 juvenile; Bocas del Toro, Cayo Solarte; 9.3336°N, 82.2189°W; depth 0.5 m; among hydroids and algae; 7 Aug 2005; T.A. Haney leg.; GCRL 6677 • 1 ♀; Bocas del Toro, Crawl Caye; depth 1–4 m; mangrove root scraping; 25 June 2023; K.N. White leg.; USNM 1762921.

**Figure 9.**
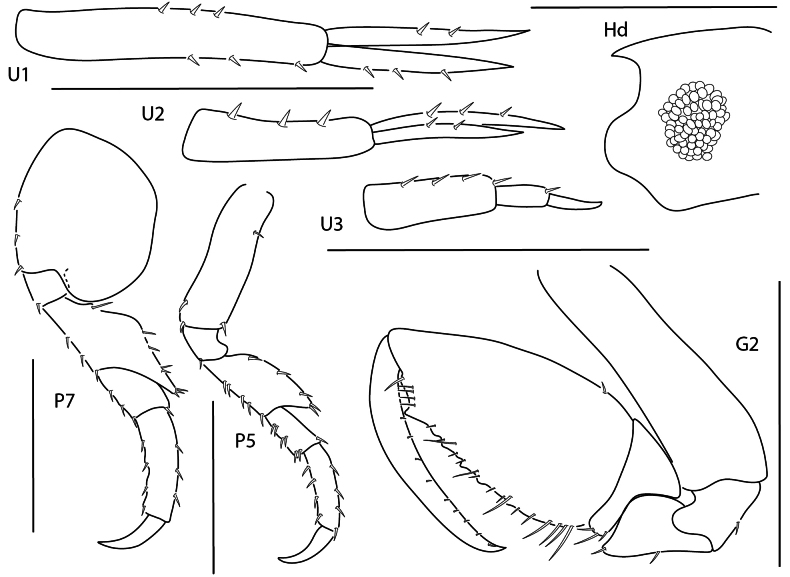
*Stenothoe valida*, female, 2.5 mm, uropods 1–3, head, pereopod 7, pereopod 5, gnathopod 2 lateral. Scale bars: 0.5 mm.

##### Diagnosis.

Head anterior margin rounded, eye relatively large. Gnathopod 2 of female propodus subovate, palmar angle absent, palm crenulate. Gnathopod 2 of male propodus palmar angle absent, with distal palmar tooth (not illustrated). Pereopod 7 merus posterodistal lobe nearly reaching distal margin of carpus. Uropod 1 peduncle lacking distoventral spur; inner ramus with two spine-setae. Uropod 3 second article of ramus subequal in length with first article of ramus.

##### Distribution.

This species has been reported as nearly cosmopolitan in tropical and warm temperate seas. Western Atlantic distribution: USA: North Carolina (Fox and Bynum 1975), South Carolina (LeCroy 2011), Biscayne Bay, Florida (LeCroy 2011), the Dry Tortugas, Florida (Pearse 1932); Bonaire: Bonaire, Kralendijk (Krapp-Schickel 2015); Bermuda: Harrington Sound (Krapp-Schickel 2015); Colombia: Barú (Ortiz and Lemaitre 1994); Brazil: Rio de Janeiro (Dana 1853; Wakabara and Serejo 1998); Panama: Bocas del Toro (present study).

##### Ecology and remarks.

This species occurs among algae, hydroids, and fouling organisms in Panama up to depths of 4 m. Previous reports list this species to depths of 33 m (LeCroy 2011). Panamanian specimens agree closely with previous descriptions of this species from the Western Atlantic (Stebbing 1906; LeCroy 2011; Krapp-Schickel 2015) and can be distinguished by the elongate merus of pereopod 7. Color of living specimens is unknown.

### ﻿Superfamily Leucothoidea Dana, 1852

**Family Leucothoidae Dana, 1852**

#### ﻿Genus Anamixis

Taxon classificationAnimaliaAmphipodaLeucothoidae

Stebbing, 1897

EBB4784B-A9DE-5251-B4A3-86D1D2224727

##### Diagnosis.

Extreme sexual dimorphism. Anamorph male. Head anterior margin oblique or rounded; mouthparts reduced. Gnathopod 1 carpochelate; coxa 1 greatly reduced, hidden by coxa 2. Gnathopod 2 enlarged, subchelate; propodus elongate with single mediofacial setal row. Female and leucomorph male. Head anterior margin transverse, mouthparts well developed. Gnathopod 2 subchelate; propodus subtriangular with transverse palm.

#### ﻿ Anamixis cavatura

Taxon classificationAnimaliaAmphipodaLeucothoidae

Thomas, 1997

FCC46AFC-FD69-5E6E-98B1-19BE1398B567

Figs 10, 23A, B

Leucothoides pottsi : Shoemaker 1933: 249–250, fig. 3; Ledoyer 1967: 127, fig. 5b; Ruffo 1969: 12–13; Sivaprakasam 1967(1969): 373, fig. 1e–g; Ledoyer 1978a: 375; Ledoyer 1978b: 300–301; Ledoyer 1979a: 169; Ledoyer 1979b: 111, fig. 68(II); Thomas 1979: 107–109; Barnard 1979: 130.
Anamixis pottsi : Ortiz and Lemaitre 1994: 124.
Anamixis hanseni : Pearse 1912: 370; Thomas 1979: 107–109; Thomas and Taylor 1981: 462–467, figs 1–5; Thomas and Barnard 1983: 154–157 [not A. hanseni Stebbing, 1897].
Anamixis cavatura Thomas, 1997: 47–50, figs 3, 4; LeCroy 2007: 506, fig. 447; White 2011: 25, fig. 1.

##### Material examined.

Panama • 1.7–4.9 mm • 2 anamorph ♂; Bocas del Toro, Cayo Solarte; 9.3336°N, 82.2189°W; depth 2 m; among red algae and *Halimeda*; 07 Aug 2005; R. Collin, M. Faust, E. Gaiser, S. LeCroy, S. Richardson, M. Sorenson leg.; GCRL 6678 • 1 leucomorph ♀; Bocas del Toro, mangrove island next to Isla Solarte; depth 2 m; among coral rubble; 08 Aug 2021; K.N. White leg.; USNM 1762922 • 1 leucomorph ♀; Bocas del Toro, Drago Beach; 9.4181°N, 82.3375°W; depth 2–3 m; among red algae; 09 Aug 2021; K.N. White leg.; USNM 1762923 • 1 leucomorph ♀; Bocas del Toro, San Cristobal; 9.2625°N, 82.2350°W; depth 15 m; among coral rubble; 10 Aug 2021; K.N. White leg.; USNM 1762924 • 1 leucomorph ♂, 2 leucomorph ♀; Bocas del Toro, Crawl Caye; 9.2376°N, 82.1438°W; depth 1.5–3 m; among *Halimeda*; 11 Aug 2021; K.N. White leg.; USNM 1762925 • 1 anamorph ♂, 1 leucomorph ♂, 16 leucomorph ♀; Bocas del Toro, Crawl Caye; 9.2475°N, 82.1290°W; depth 5 m; among coral rubble; 12 Aug 2021; K.N. White leg.; USNM 1762926 • 1 anamorph ♂; Bocas del Toro, Hospital Point, 9.3319°N, 82.2148°W; depth 1–3 m; among *Halimeda*; 22 June 2023; K.N. White leg.; USNM 1762927 • 2 anamorph ♂; Bocas del Toro, Crawl Caye; 9.2459°N, 82.1369°W; depth 1–4 m; among coral rubble and *Halimeda*; 25 June 2023; K.N. White leg.; USNM 1762928 • 1 anamorph ♂; Bocas del Toro, Cayo Zapatilla 1; 9.2699°N, 82.0587°W; depth 10–11 m; among coral rubble; 28 June 2023; K.N. White leg.; USNM 1762929.

**Figure 10.**
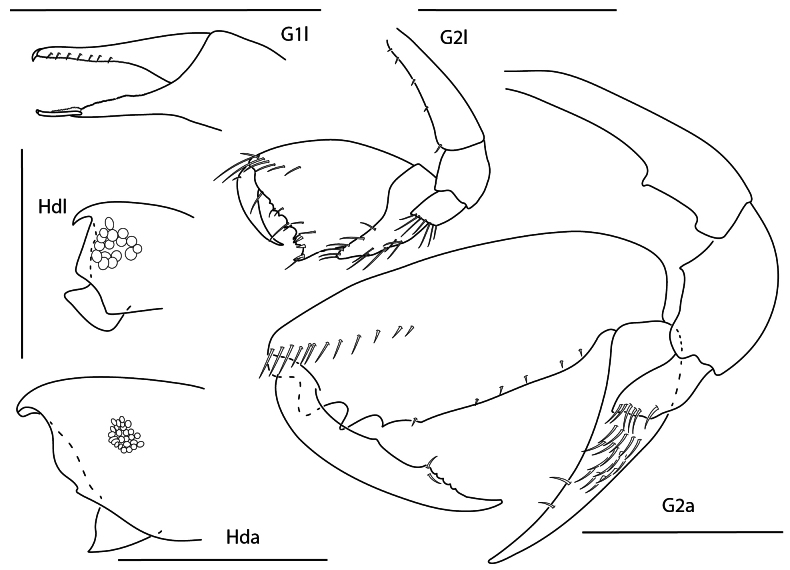
*Anamixis cavatura*, leucomorph female (l), 3 mm, gnathopod 1 medial, gnathopod 2 medial, head; anamorph male (a), 3.1 mm, head, gnathopod 1 medial, gnathopod 2 medial; Scale bars: 0.5 mm.

##### Diagnosis.

Anamorph male. Head anterior margin with small tooth-like projection; ventral keel elongate, subtriangular with pointed tip. Gnathopod 2 basis anteromedial margin with acute projection; propodus palm with several teeth; dactylus with distal serrations.

Leucomorph male and female. Head anterior margin quadrate; ventral keel subrectangular. Gnathopod 1 carpus finely serrate, apex with two serrate spines, larger spine with bifid tip. Gnathopod 2 palm transverse, irregularly serrate.

##### Distribution.

USA: Dry Tortugas, Florida (Shoemaker 1933), Fort Pierce to Tampa, including the Florida Keys (Thomas 1997; LeCroy 2007), Mississippi Delta (Pearse 1912); Colombia: Isla Barú (Ortiz and Lemaitre 1994); Bahamas; Mexico: Yucatan; Belize: Carrie Bow Caye; Cuba: Guanahacabibes (Varela et al. 2003); Venezuela (Martín et al. 2013); Honduras; Jamaica; Greater and Lesser Antilles (Thomas 1997); Panama: Bocas del Toro (White 2011; present study).

##### Ecology and remarks.

This species occurs among red algae, *Halimeda*, and coral rubble at depths of 1–11 m. Panamanian anamorph males differ from the original description in having the gnathopod 2 basis and ischium bare (vs each with one seta) and dactylus distally serrate (vs entire margin serrate). Living anamorphs are translucent white with brown splotches and have a red eye. Living leucomorphs are translucent white with magenta stripes.

#### ﻿ Anamixis vanga

Taxon classificationAnimaliaAmphipodaLeucothoidae

Thomas, 1997

88595FD8-FB78-5418-A7A8-5F7B93554574

Figs 11, 23C, D

Anamixis vanga Thomas, 1997: 70–73, figs 17, 18; LeCroy 2007: 507, fig. 448; White 2011: 25–26, fig. 2.

##### Material examined.

Panama • 1–4 mm • 1 leucomorph ♂, 1 leucomorph ♀, 1 leucomorph juvenile; Bocas del Toro, Mangrove Inn; among coral rubble; 03 Aug 2005; M. Faust, E. Gaiser, T. Haney, S. Richardson, M. Sorenson leg.; GCRL 6679 • 3 anamorph ♂, 2 leucomoprh ♂, 6 leucomorph ♀; Bocas del Toro, Hospital Point; 9.3336°N, 82.2188°W; depth 15 m; among coral rubble and *Halimeda*; 06 Aug 2005; S. DeGrave, M. Salazar leg.; GCRL 6680 • 3 anamorph ♂, 2 leucomorph♀; Bocas del Toro, Cayo Solarte; 9.3336°N, 82.2189°W; depth 2 m; among sponges, coral rubble, and sand; 07 Aug 2005; R. Collin, M. Faust, E. Gaiser, S. LeCroy, S. Richardson, M. Sorenson leg.; GCRL 6681 • 1 anamorph ♂, 2 leucomorph ♂, 6 leucomorph ♀; Bocas del Toro, Pigeon Key Reef; 9.2693°N, 82.2489°W; depth 0.5–1 m; among *Halimeda* and *Thalassia*; 09 Aug 2005; E. Baiser, T.A. Haney, S. LeCroy leg.; GCRL 6682 • 1 leucomorph ♀; Bocas del Toro, Isla Solarte; 9.2901°N, 82.1897°W; depth 1–5 m; among *Halimeda*; 08 Aug 2021; K.N. White leg.; USNM 1762930 • 2 leucomorph ♀; Bocas del Toro, Drago; 9.4181°N, 82.3375°W; depth 2–3 m; among coral rubble; 09 Aug 2021; K.N. White leg.; USNM 1762931 • 1 leucomorph ♀; Bocas del Toro, Pandora; 9.3278°N, 82.2222°W; depth 10 m; among coral rubble; 10 Aug 2021; K.N. White leg.; USNM 1762932 • 1 leucomorph ♀; Bocas del Toro, San Cristobal; 9.2849°N, 82.2945°W; depth 1–3 m; among *Dictyota*; 21 June 2023; K.N. White leg.; USNM 1762933 • 1 anamorph ♂, 1 leucomorph ♂, 6 leucomorph ♀; Bocas del Toro, Hospital Point; 9.3319°N, 82.2148°W; depth 1–3 m; among coral rubble; 22 June 2023; K.N. White leg.; USNM 1762934 • 2 leucomorph ♀; Bocas del Toro, Drago; 9.4134°N, 82.3334°W; depth 1–3 m; among coral rubble; 23 June 2023; K.N. White leg.; USNM 1762935 • 1 leucomorph ♀; Bocas del Toro, Swan Caye; 9.4536°N, 82.3000°W; depth 2 m; among coral rubble; 24 June 2023; K.N. White leg.; USNM 1762936 • 2 anamorph ♂, 1 leucomorph ♂, 4 leucomorph ♀; Bocas del Toro, Crawl Caye; 9.2476°N, 82.1290°W; depth 5–8 m; among coral rubble; 26 June 2023; K.N. White leg.; USNM 1762937 • 1 anamorph ♂; Bocas del Toro, Pandora; 9.3333°N, 82.2185°W; depth 7 m; among coral rubble; 26 June 2023; K.N. White leg.; USNM 1762938 • 2 leucomorph ♀; Bocas del Toro, STRI Dock; 9.3512°N, 82.2570°W; depth 0–1 m; dock scraping; 27 June 2023; K.N. White leg.; USNM 1762939 • 1 leucomorph ♂, 3 leucomorph ♀; Bocas del Toro, Cayo Zapatilla 1; 9.2699°N, 82.0587°W; depth 10–11 m; among coral rubble; 28 June 2023; K.N. White leg.; USNM 1762940 • 1 anamorph ♂, 6 leucomorph♀; Bocas del Toro, Crawl Caye; 9.2502°N, 82.1318°W; depth 5–13 m; among coral rubble; 29 June 2023; K.N. White leg.; USNM 1762941.

**Figure 11.**
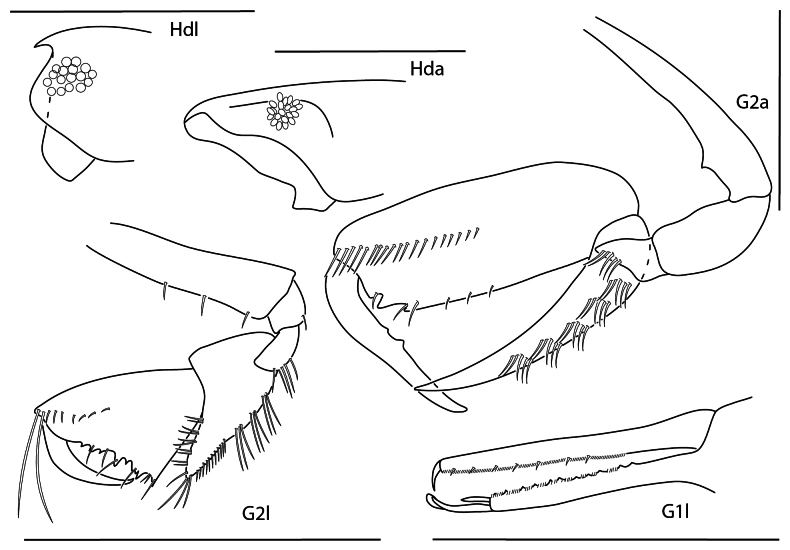
*Anamixis vanga*, leucomorph female (l), 2.2 mm, head, gnathopod 2 medial, gnathopod 1 medial; anamorph male (a), 3.2 mm, head, gnathopod 2 medial. Scale bars: 0.5 mm.

##### Diagnosis.

Anamorph male. Head with lateral ridge, anterior margin concave, without anterodistal notch, ventral keel rectangular. Gnathopod 2 basis with acute process subdistally; propodus palm with three teeth.

Leucomorph male and female. Head ocular lobe rounded; ventral keel rounded, subacute distally. Gnathopod 1 propodus margin finely serrate with several small setae; carpus dentate and finely serrate, terminal spines smooth with terminal bulb. Gnathopod 2 propodus palm oblique with several serrations.

##### Distribution.

USA: Georgia to the Florida Keys (Thomas 1997); Belize: Carrie Bow Caye (Thomas 1997); Mexico (Martín et al. 2013); Venezuela (Martín et al. 2013); Panama: Bocas del Toro (White 2011; present study).

##### Ecology and remarks.

This species occurs among sponges, *Halimeda*, *Thalassia*, and coral rubble at depths of 1–11 m. Thomas (1997) reported this species from solitary ascidians at depths to 20 m. Panamanian specimens agree closely with previous descriptions of the species. Anamorph males have an anterior tubercle on the gnathopod 2 basis that was not illustrated in original description as noted by White (2011). Living anamorphs and leucomorphs are translucent with pale brown stripes and have red eyes.

#### ﻿Genus Leucothoe

Taxon classificationAnimaliaAmphipodaLeucothoidae

Leach, 1814

86BF29D0-6C47-5B28-A210-3D7E8A04E30C

##### Diagnosis.

Minimal sexual dimorphism. Eyes well-developed. Mandibular palp tri-articulate; left lacinia mobilis larger than right. Maxilliped outer plate not longer than palp article 1. Coxa 1–4 subequal in size. Gnathopod 1 carpochelate. Pereopods 5–7 bases expanded.

#### ﻿ Leucothoe alata

Taxon classificationAnimaliaAmphipodaLeucothoidae

Barnard, 1959

95A77939-FDD5-5C01-A495-759F0A75866F

Figs 12, 23E

Leucothoe minima Barnard, 1952: 9–12, pl. 1 [not Schellenberg, 1925: 141–142, fig. 12].
Leucothoe alata Barnard 1959: 19–20, pl. 1; Barnard 1962: 132, figs 7D–F; Barnard 1964a: 114; Barnard 1964b: 227; Barnard 1966: 22; Barnard 1969a: 164; Barnard 1969b: 214; Barnard 1979: 128–129; Hirayama 1985: 172–174, figs 166, 169.

##### Material examined.

Panama • 2.5–3.2 mm • 1 ♂, 1 ♀; Bocas del Toro, Cayo Zapatilla; 9.2699°N, 82.0587°W; depth 0 m; buoy scraping; 29 June 2023; K.N. White leg.; USNM 1762942.

**Figure 12.**
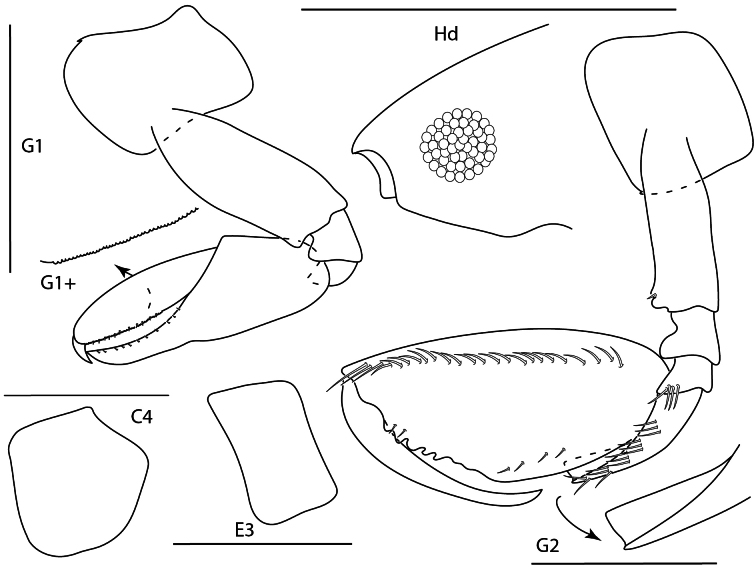
*Leucothoe alata*, male, 2.5 mm, gnathopod 1 medial, head, coxa 4, epimeron 3, gnathopod 2 medial. Scale bars: 0.5 mm.

##### Diagnosis.

Head anterodistal margin subquadrate without cusp; ventral keel slightly produced. Gnathopod 1 basis anterior and posterior margins bare; carpus thickened; propodus expanded, palm dentate; dactylus reaching less than 0.2 × propodus length. Gnathopod 2 basis with anterodistal projection; carpus distally truncate, spoon-like; propodus with one mediofacial setal row above midline, reaching 0.9 × propodus length, palm with several small tubercles. Coxa 4 posterior margin slightly concave. Epimeron 3 posteroventral corner subquadrate.

##### Distribution.

USA: Monterey Bay to San Ramon Bay, California (Barnard 1952, 1955, 1962, 1964a, 1964b, 1966, 1969a, 1969b, 1979); Japan: West Kyushu, Tomioka Bay (Hirayama 1985); Panama: Bocas del Toro (present study).

##### Ecology and remarks.

This species occurs among sponges on docks (Barnard 1959) and buoys, suggesting that this species may be easily transported with fouling organisms. This may partially explain the range extension of this species to include the Caribbean Sea, when it was previously only documented from the Pacific Ocean. Panamanian specimens agree closely with the original description of this species, except for the anterodistal head margin appearing slightly more truncated in Panamanian specimens. The color of living specimens is unknown.

#### ﻿ Leucothoe ashleyae

Taxon classificationAnimaliaAmphipodaLeucothoidae

Thomas & Klebba, 2006

66C12B57-6D4D-5268-960F-BF30AD38E64B

Figs 13, 23F

Leucothoe ashleyae Thomas & Klebba, 2006: 14–16, figs 1–3, 7; LeCroy 2011: 626, fig. 515; White 2011: 26–27, fig. 3.

##### Material examined.

Panama • 1–3 mm • 2 ♂; Bocas del Toro, STRI Point; 07 Aug 2005; S. DeGrave, M. Salazar leg.; GCRL 6683 • 4 juveniles; Bocas del Toro, Bastamientos; 07 Aug 2005; T.A. Haney leg.; GCRL 6684 • 1 ♀; Bocas del Toro, STRI Point; 9.3487°N, 82.2626°W; depth 12 m; among coral rubble; 06 Aug 2021; K.N. White leg.; USNM 1762943 • 2 ♂, 7 ♀; Bocas del Toro, Almirante; 9.2900°N, 82.3429°W; depth 10–11 m; commensal in *Lissodendoryx columbiensis*; 07 Aug 2021; K.N. White leg.; USNM 1762944 • 1 ♂, 1 ♀; Bocas del Toro, Swan Caye; 9.4536°N, 82.3000°W; depth 2 m; in sand; 24 June 2023; K.N. White leg.; USNM 1762945 • 4 ♂, 6 ♀, 3 juveniles; Bocas del Toro, Punta Caracol; depth 1–2 m; commensal in *Lissodendoryx columbiensis* and among *Halimeda*; 24 June 2023; K.N. White leg.; USNM 1762946 • 1 ♀; Bocas del Toro, Crawl Caye; 9.2476°N, 82.1290°W; depth 5–8 m; among coral rubble; 26 June 2023; K.N. White leg.; USNM 1762947 • 1 ♀; Bocas del Toro, Pandora; 9.3333°N, 82.2185°W; depth 7 m; among coral rubble; 26 June 2023; K.N. White leg.; USNM 1762948.

**Figure 13.**
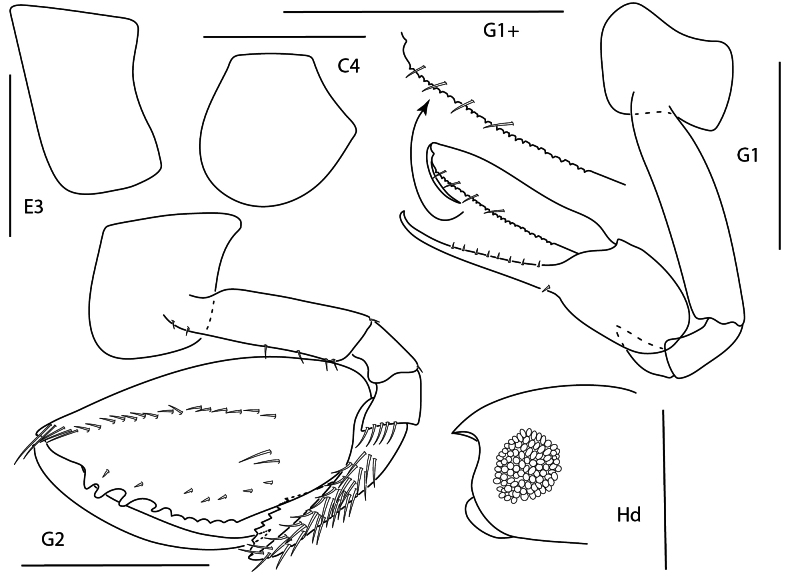
*Leucothoe ashleyae*, female, 3.6 mm, epimeron 3, coxa 4, gnathopod 1 medial, gnathopod 2 medial, head. Scale bars: 0.5 mm.

##### Diagnosis.

Head anterodistal margin rounded; ventral keel rounded. Gnathopod 1 basis anterior margin with 0–1 seta, posterior margin bare; propodus palm serrate; dactylus reaching more than 0.2 × propodus length. Gnathopod 2 basis anterior margin with six short setae; carpus distally expanded, anterior margin dentate; propodus primary mediofacial setal row above midline, reaching 0.7 × propodus length, secondary mediofacial row with two or three setae, submarginal setal row present, palm with several small tubercles. Coxa 4 posterior margin slightly concave. Epimeron 3 posteroventral corner subquadrate.

##### Distribution.

USA: South Florida, Florida Keys; Bahamas; Puerto Rico: Vieques; Mexico (Martín et al. 2013); Belize: Whale Shoals; Honduras: Roatan (Thomas and Klebba 2006); Panama: Bocas del Toro (White 2011; present study).

##### Ecology and remarks.

This species occurs as a commensal in sponges and among coral rubble and algae at depths of 1–12 m. Panamanian specimens agree closely with previous descriptions of the species, with the exception of having gnathopod 1 coxa anterodistal corner smooth (vs serrate), carpus with distal setae (vs bare), and variation in the number of setae on gnathopod 2, remaining coxae, and pereopod 5–7 bases as noted by White (2011). Living specimens are translucent orange with a red eye.

#### ﻿ Leucothoe barana

Taxon classificationAnimaliaAmphipodaLeucothoidae

Thomas & Klebba, 2007

E967EF93-FAC2-5093-B480-3A3A332A03C7

Figs 14, 23G

Leucothoe barana Thomas & Klebba, 2007: 5–10, figs 1–3, 7A, B; LeCroy 2011: 628, fig. 519; White 2011: 27, 28, fig. 4.

##### Material examined.

Panama • 2–5 mm • 1 ♂, 1 ♀; Bocas del Toro, Crawl Cay; 9.2508°N, 82.1311°W; depth 0–2 m; commensal in *Tedania* sponge; 1 Aug 2005; J.D. Thomas leg.; GCRL 6685 • 1 ♀; Bocas del Toro, Swan Caye; 9.4533°N, 82.2148°W; depth 3 m; commensal in orange sponge; 04 Aug 2005; T. Haney leg.; GCRL 6686 • 5 ♂, 4 ♀, 1 juvenile; Bocas del Toro, STRI Point; depth 15 m; among coral rubble and *Halimeda*; 06 Aug 2005; S. DeGrave leg.; GCRL 6687 • 2 ♀; Bocas del Toro, Cayo Solarte; 9.3044°N, 82.1316°W; depth 1.5 m; among sponges, coral rubble, and sand; 07 Aug 2005; R. Collin, M. Faust, E. Gaiser, S. LeCroy, S. Richardson, M. Sorensen leg.; GCRL 6688 • 7 ♂, 6 ♀, 9 juveniles; Bocas del Toro, Laboratory Dock; 9.3512°N, 82.2570°W; depth 0.5 m; mangrove sponge; 08 Aug 2005; T.A. Haney leg.; GCRL 6689 • 2 ♂; Bocas del Toro, STRI Point; 9.3487°N, 82.2626°W; depth 12 m; commensal in loggerhead sponge; 06 Aug 2021; K.N. White leg.; USNM 1762949 • 2 ♀; Bocas del Toro, Drago; 9.4181°N, 82.3375°W; depth 2–3 m; among coral rubble; 09 Aug 2021; K.N. White leg.; USNM 1762950 • 1 ♀, 1 juvenile; Bocas del Toro, Drago; 9.4134°N, 82.3334°W; depth 1–3 m; among sponges and coral rubble; 23 June 2023; K.N. White leg.; USNM 1762951 • 1 ♀, 1 juvenile; Bocas del Toro, Punta Caracol; depth 1–2 m; commensal in *Lissodendoryx columbiensis*; 24 June 2023; K.N. White leg.; USNM 1762952.

**Figure 14.**
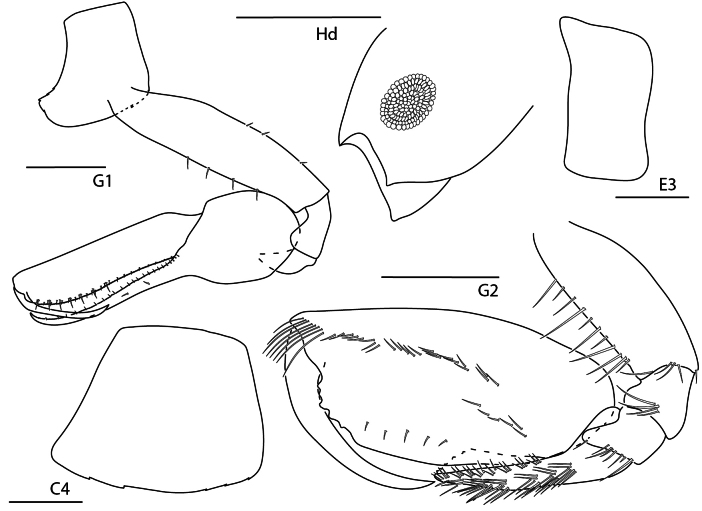
*Leucothoe barana*, female, 5.5 mm, gnathopod 1 medial, head, epimeron 3, coxa 4, gnathopod 2 medial. Scale bars: 0.5 mm.

##### Diagnosis.

Head anterodistal margin quadrate with cusp; ventral keel projected with cusp. Coxae 1–4 anterodistal and/or ventral margins serrate; coxa 4 posterior margin tapered. Gnathopod 1 basis anterior and posterior margins setose; carpus with two facial setae; propodus palm dentate; dactylus reaching greater than 0.2 × propodus length. Gnathopod 2 basis anterior margin lined with setae; carpus distally truncate; propodus mediofacial setal row reaching 0.7 × propodus length, secondary mediofacial row with six setae, palm with several tubercles. Epimeron 3 posteroventral corner narrowly rounded.

##### Distribution.

USA: Florida Keys; Belize: Pelican Cays (Thomas and Klebba 2007); Panama: Bocas del Toro (White 2011; present study).

##### Ecology and remarks.

This species occurs as a commensal in sponges and among coral rubble and algae at depths of 1–12 m. Panamanian specimens agree closely with previous descriptions of this species with the exception of coxa 4 having a smooth anterior margin (vs having two sharp cusps in original description). Living specimens are translucent purple in color with a red eye.

#### ﻿ Leucothoe flammosa

Taxon classificationAnimaliaAmphipodaLeucothoidae

Thomas & Klebba, 2007

33ECD3E2-9BB0-5839-B6D9-A3E8C8852BB0

Figs 15, 23H

Leucothoe spinicarpa Ortiz 1975: 8.
Leucothoe flammosa Thomas & Klebba, 2007: 10–15, figs 4–6, 7C, D; LeCroy 2011: 629, fig. 505; White 2011: 28–29, fig. 5.

##### Material examined.

Panama • 4.5–6.5 mm • 1 ♂, 1 ♀; Bocas del Toro, STRI Point; 9.3487°N, 82.2626°W; depth 12 m; commensal in *Lima scabra*; 06 Aug 2021; K.N. White leg.; USNM 1762953 • 1 ♂, 2 ♀; Bocas del Toro, Almirante; 9.2900°N, 82.3429°W; depth 10–11 m; commensal in *Lima scabra*; 07 Aug 2021; K.N. White leg.; USNM 1762954 • 3 ♀; Bocas del Toro, Almirante; 9.2900°N, 82.3429°W; depth 10–11 m; from ark clams; 07 Aug 2021; K.N. White leg.; USNM 1762955.

**Figure 15.**
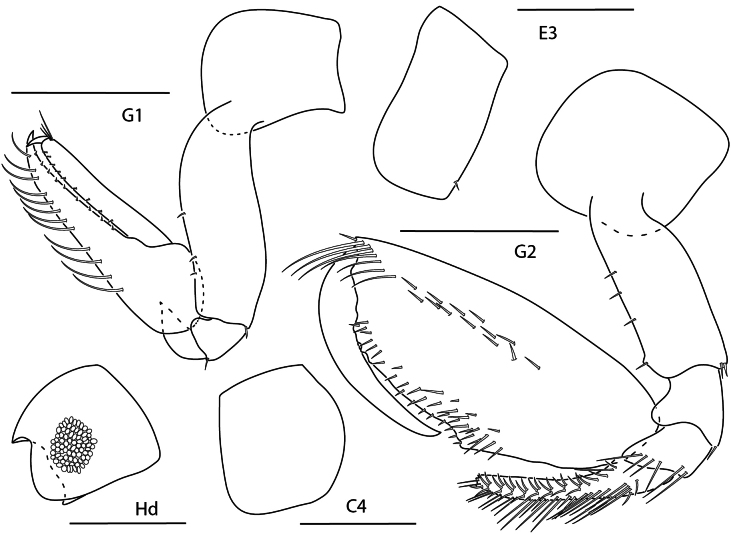
*Leucothoe flammosa*, male, 5 mm, gnathopod 1 medial, epimeron 3, head, coxa 4, gnathopod 2 medial. Scale bars: 0.5 mm.

##### Diagnosis.

Head anteroventral margin round. Coxae 1–4 smooth; coxa 4 posterior margin rounded. Gnathopod 1 basis expanded, posterior margin lacking setae; carpus expanded, posterior margin with row of long setae; propodus palm smooth; dactylus reduced, nail-like. Gnathopod 2 basis with few setae; carpus narrowing distally, tridentate; propodus mediofacial setal row above midline, reaching 0.6 × length of propodus, dense row of submarginal setae present, palm with several tubercles; dactylus reaching 0.5 × length of propodus. Epimeron 3 posteroventral margin subquadrate, with a single seta.

##### Distribution.

USA: Florida Keys (Thomas and Klebba 2007); Belize: Pelican Cays (Thomas and Klebba 2007); Cuba: Gulf of Batabanó and Baracoa Beach, Barlovento (Ortiz 1975); Panama: Bocas del Toro (White 2011; present study).

##### Ecology and remarks.

This species occurs in the mantle cavities of bivalve mollusks at depths of 1–20 m. Panamanian specimens differ from the original description in being generally less setose (specifically on coxae and epimera) and lacking setae on gnathopod 1 basis posterior margin. White (2011) illustrated specimens of *L. flammosa* from Panama with fewer shorter setae on the gnathopod 1 basis posterior margin, suggesting that this character may vary within the species. Living specimens are translucent white with brown eyes. Ommatidia separated with white coloration. Females have yellow-green eggs.

#### ﻿ Leucothoe kensleyi

Taxon classificationAnimaliaAmphipodaLeucothoidae

Thomas & Klebba, 2006

7D40CF7E-BBC1-523E-B58C-7052A94E7FED

Figs 16, 24A

Leucothoe tridens : Barnard 1965: 492; 1970: 211, fig. 137; 1971: 103; Serejo 1998: 109–112, figs 3, 4 [not Leucothoe tridens Stebbing, 1888].
Leucothoe kensleyi Thomas & Klebba, 2006: 17–22, figs 4–7; LeCroy 2011: 631, fig. 509; White 2011: 29–30, fig. 6.

##### Material examined.

Panama • 4–5.6 mm • 1 ♀; Bocas del Toro, Juan Point; 9.3015°N, 82.2940°W; depth 10 m; among coral rubble; 07 Aug 2021; K.N. White leg.; USNM 1762956 • 1 ♂; Bocas del Toro, Drago 9.4181°N, 82.3375°W; depth 2–3 m; among coral rubble; 09 Aug 2021; K.N. White leg.; USNM 1762957 • 1 ♂, 1 ♀, 1 juvenile; Bocas del Toro, Hospital Point; 9.3319°N, 82.2148°W; depth 1–3 m; among coral rubble; 22 June 2023; K.N. White leg.; USNM 1762958 • 4 ♀; Bocas del Toro, Crawl Caye; 9.2459°N, 82.1369°W; depth 1–4 m; among coral rubble; 25 June 2023; K.N. White leg.; USNM 1762959.

**Figure 16.**
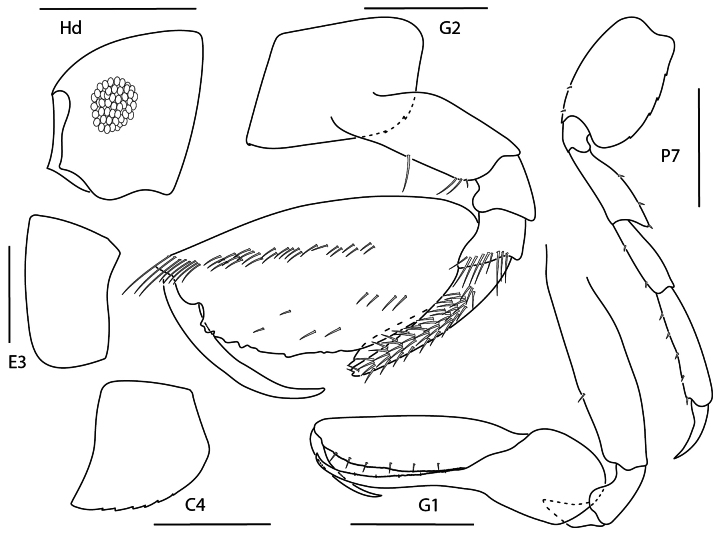
*Leucothoe kensleyi*, male, 4.8 mm, head, gnathopod 2 medial, epimeron 3, coxa 4, gnathopod 1 medial, pereopod 7. Scale bars: 0.5 mm.

##### Diagnosis.

Head anterodistal margin quadrate with cusp; ventral keel acute. Coxae 1–3 each with antero- and posterodistal cusp, coxa 4 ventrally serrate, posteriorly tapered. Gnathopod 1 basis anterior margin with few setae, posterior margin bare; carpus with one facial seta; propodus palm dentate; dactylus reaching greater than 0.2 × propodus length. Gnathopod 2 basis anterior margin with few setae; carpus tapering distally; propodus mediofacial setal row above midline, reaching 0.7 × propodus length, secondary mediofacial row with 2–4 setae, palm with several small tubercles. Pereopod 5–7 bases narrowly expanded. Epimeron 3 posteroventral corner quadrate.

##### Distribution.

USA: Hawaii (Thomas and Klebba 2006), South Carolina (LeCroy 2011), South Florida to the Florida Keys (Thomas and Klebba 2006); Mexico (Martín et al. 2013); Belize: Carrie Bow Cay (Thomas and Klebba 2007); Turks and Caicos Islands (LeCroy 2011); Brazil: Albrolhos (Thomas and Klebba 2006); Panama: Bocas del Toro (White 2011; present study).

##### Ecology and remarks.

This species occurs among sponges and coral rubble from 1–20 m. Panamanian specimens agree closely with previous descriptions by Thomas and Klebba (2006) and White (2011) with the exception of coxa 4 having a smooth anterior margin and a slightly rounded posteroventral corner and a smooth ventral margin on epimeron 3. Living specimens are translucent pink in color with purple coloration on dorsal surface and red eyes.

#### ﻿ Leucothoe laurensi

Taxon classificationAnimaliaAmphipodaLeucothoidae

Thomas & Ortiz, 1995

89ADF42E-5022-54FE-9BF4-2D3C1062B313

Figs 17, 24B

Leucothoe laurensi Thomas & Ortiz, 1995: 613–616, figs 1, 2; Serejo 1998: 117–119, fig. 8; LeCroy 2011: 632, fig. 507; White 2011: 30, fig. 7.

##### Material examined.

Panama • 2.4–3.2 mm • 2 ♂, 5 ♀; Bocas del Toro, Hospital Point; 9.3336°N, 82.2188°W; depth 15 m; among coral rubble and *Halimeda*; 6 Aug 2005; S. DeGrave, M. Salazar leg.; GCRL 6690 • 8 ♀; Bocas del Toro, Crawl Caye; 9.2459°N, 82.1369°W; depth 1–4 m; among coral rubble; 25 June 2023; K.N. White leg.; USNM 1762960 • 1 ♂, 6 ♀; Bocas del Toro, Crawl Caye; 9.2476°N, 82.1290°W; depth 5–8 m; among coral rubble; 26 June 2023; K.N. White leg.; USNM 1762961.

**Figure 17.**
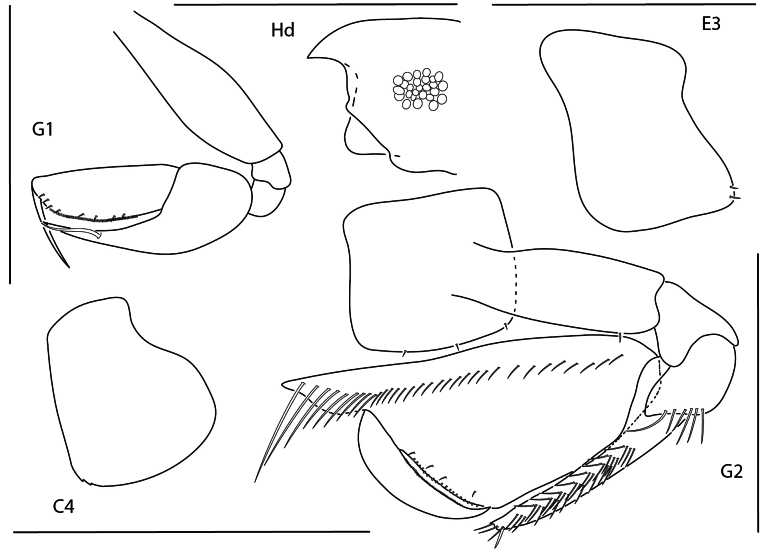
*Leucothoe laurensi*, female, 2.3 mm, gnathopod 1 medial, head, epimeron 3, coxa 4, gnathopod 2 medial. Scale bars: 0.5 mm.

##### Diagnosis.

Head anterior margin oblique with rounded cusp; ventral keel rounded. Gnathopod 1 basis distally expanded, margins bare; carpus expanded, proximal margin with long distal seta; propodus inflated; dactylus reaching greater than 0.2 × propodus length. Gnathopod 2 carpus elongate, distally truncate; propodus distal margin with blade-like process, mediofacial row above midline, reaching almost entire length of propodus, palm subrectangular; dactylus short, thick. Coxa 4 anteroventral margin serrate, posterior margin excavate. Epimeron 3 posteroventral corner rounded with two setae.

##### Distribution.

USA: North Carolina to the Florida Keys (Thomas and Ortiz 1995), South Carolina (LeCroy 2011), Cuba: Isla de la Juventud (Thomas and Ortiz 1995); Brazil: Pernambuco, Alagoas (Serejo 1998); Turks and Caicos Islands (LeCroy 2011); Panama: Bocas del Toro (White 2011; present study).

##### Ecology and remarks.

This species occurs among coral rubble and fine sand from 1–50 m (Thomas and Ortiz 1995). Panamanian specimens agree with previous descriptions of this species with the exception of lacking a secondary mediofacial setal row on gnathopod 2 (vs a secondary row with one seta). Living specimens are translucent white with magenta stripes.

#### ﻿ Leucothoe machidai

Taxon classificationAnimaliaAmphipodaLeucothoidae

White, 2019

32CBA042-6E72-5C4D-AA32-C0773BC01A6C

Figs 18, 24C

Leucothoe machidai White, 2019: 5–8, figs 4, 5.

##### Material examined.

Panama • 2–6.5 mm • 2 ♀; Bocas del Toro, Hospital Bight; 9.3044°N, 82.1316°W; depth 1.5 m; among sponges, coral rubble, and sand; 7 Aug 2005; T.A. Haney leg.; 1GCRL 669 • 1 ♀; Bocas del Toro, Cayo Solarte; 9.3336°N, 82.2189°W; depth 2 m; among coral rubble; 7 Aug 2005; R. Collin, M. Faust, E. Gaiser, S. LeCroy, S. Richardson, M. Sorensen leg.; GCRL 6692 • 1 ♂, 1 ♀; Bocas del Toro, Drago; 9.4134°N, 82.3334°W; depth 1–3 m; among coral rubble; 23 June 2023; K.N. White leg.; USNM 1762962 • 1 ♂, 2 ♀; Bocas del Toro, Crawl Caye; 9.2476°N, 82.1290°W; depth 5–8 m; among coral rubble; 26 June 2023; K.N. White leg.; USNM 1762963 • 3 ♂, 5 ♀; Bocas del Toro, Crawl Caye; depth 0–1 m; buoy scraping; 28 June 2023; K.N. White leg.; USNM 1762964.

**Figure 18.**
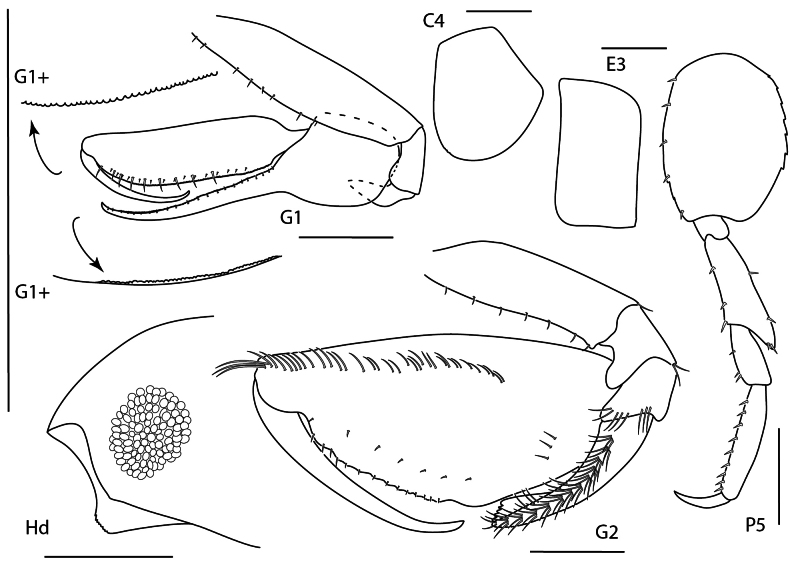
*Leucothoe machidai*, male, 6 mm, gnathopod 1 medial, coxa 4, epimeron 3, head, gnathopod 2 medial, pereopod 5. Scale bars: 0.5 mm.

##### Diagnosis.

Head anterior margin truncate; ventral keel anterior margin weakly concave, anteroventral margin serrate. Gnathopod 1 basis anterior margin with a few short setae; carpus proximal margin dentate, lacking facial setae; propodus palm dentate, with distal setae; dactylus reaching greater than 0.2× propodus length. Gnathopod 2 basis anterior margin with several short setae; carpus distally rounded, anterior margin dentate; propodus primary mediofacial setal row above midline, slightly displaced, reaching 0.7 × propodus length, secondary row with three setae, palm with small tubercles, one row of submarginal setae present. Coxa 4 minutely serrate, posterior margin tapered. Epimeron 3 posteroventral corner quadrate.

##### Distribution.

USA: Tampa Bay, Florida (White 2019); Panama: Bocas del Toro (present study).

##### Ecology and remarks.

This species occurs among sponges, sand, and coral rubble and on buoys in Panama at depths of 0–8 m. White (2019) recorded this species from solitary ascidians and sponges in Tampa, Florida. Panamanian specimens agree closely with the original description, with the exception of the anterior head margin being more truncate than rounded. Living specimens are translucent ivory with a pink tint.

#### ﻿ Leucothoe tunica

Taxon classificationAnimaliaAmphipodaLeucothoidae

White, 2019

949A5F40-5CA0-5B49-947F-53A81914057B

Figs 19, 24D

Leucothoe n. sp. C: Thomas and Klebba 2007: 40 fig. 25C.
Leucothoe n. sp. B: Thomas and Klebba 2007: 41, tab. 4 (not Leucothoe n. sp. B: Thomas and Klebba 2007: 40, fig. 25B).
Leucothoe sp. B: LeCroy 2011: 636, fig. 516.
Leucothoe tunica White, 2019: 2–5, figs 1, 2.

##### Material examined.

Panama • 2–5.9 mm • 1 ♂, 2 ♀; Bocas del Toro, Swan Caye; 9.4533°N, 82.2983°W; depth 3 m; commensal in orange sponge; 4 Aug 2005; M. Faust, E. Gaiser, T. Haney, S. Richardson, M. Sorenson leg.; GCRL 6693 • 1 ♀; Bocas del Toro, Juan Point; 9.3015°N, 82.2940°W; depth 10 m; among coral rubble; 7 Aug 2021; K.N. White leg.; USNM 1762965 • 2 ♂, 5 ♀, 2 juveniles; Bocas del Toro, Drago; 9.4131°N, 82.3334°W; depth 1–3 m; among sponges and coral rubble; 23 June 2023; K.N. White leg.; USNM 1762966 • 1 ♂; Bocas del Toro, Punta Caracol; depth 1–2 m; among *Halimeda*; 24 June 2023; K.N. White leg.; USNM 1762967 • 1 ♀; Bocas del Toro, Crawl Caye; 9.2459°N; 82.1369°W; depth 1–4 m; among coral rubble; 25 June 2023; K.N. White leg.; USNM 1762968.

**Figure 19.**
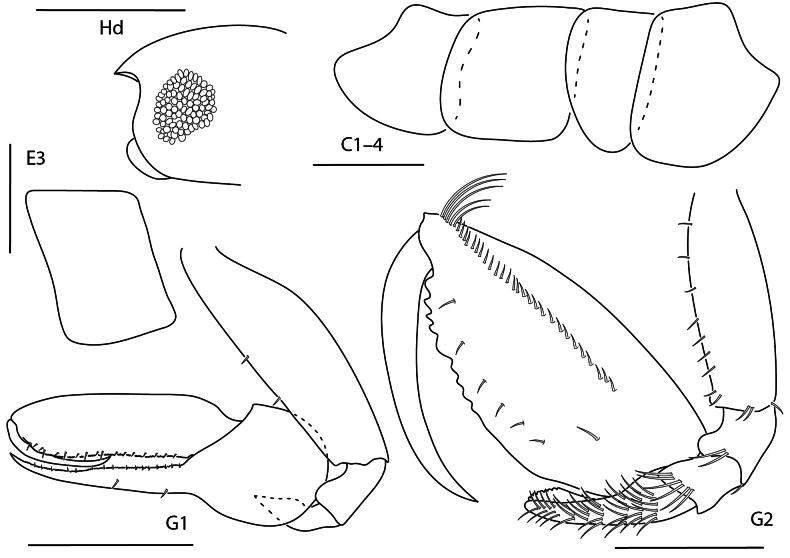
*Leucothoe tunica*, male, 5.2 mm, head, coxae 1–4, epimeron 3, gnathopod 1 medial, gnathopod 2 medial. Scale bars: 0.5 mm.

##### Diagnosis.

Head anterior margin rounded; ventral keel rounded. Gnathopod 1 basis anterior margin with two short setae; carpus proximal margin dentate, with two facial setae; propodus palm dentate, with distal setae; dactylus reaching greater than 0.2 × propodus length. Gnathopod 2 basis anterior margin with several short setae; carpus distally rounded, anterior margin dentate; propodus primary mediofacial setal row above midline, slightly displaced, reaching 0.7× propodus length, secondary row with one seta, palm with several tubercles, one row of submarginal setae present. Coxa 4 smooth, posterior margin weakly concave. Epimeron 3 posteroventral corner subquadrate.

##### Distribution.

USA: Tampa Bay and Tarpon Springs, Florida (White 2019), south Florida to the Florida Keys (Thomas and Klebba 2007), Apalachee Bay, Florida (LeCroy 2011), Mobile Bay, Alabama (LeCroy 2011), 7 ½ Fathom Reef, Texas (LeCroy 2011), and South Carolina (LeCroy 2011); Belize: Carrie Bow Cay (Thomas and Klebba 2007); Panama, Bocas del Toro (White 2011; this study).

##### Ecology and remarks.

This species occurs among sponges, *Halimeda*, and coral rubble at depths of 1–11 m. White (2019) reported this species as commensal in several species of sponges, ascidians at depths to 15 m. Panamanian specimens agree closely with the original description, with the exception of the following: gnathopod 1 basis anterior margin with 2 setae (vs 4–6 setae), posterior margin bare (vs 2–5 setae); a second mediofacial row of 1–3 setae on gnathopod 2 propodus (vs lacking) and a weakly concave posterior margin on coxa 4 (vs tapered). Living specimens are translucent ivory to pink in color.

#### ﻿ Leucothoe ubouhu

Taxon classificationAnimaliaAmphipodaLeucothoidae

Thomas & Klebba, 2007

62ADF250-2811-54F4-B966-EF3FF4207C26

Figs 20, 24E

Leucothoe ubouhu Thomas & Klebba, 2007: 25–30, figs 15–18, 22A, B; LeCroy 2011: 633, fig. 521; White 2011: 30–31, fig. 8.

##### Material examined.

Panama • 1–7 mm • 1 juvenile; Bocas del Toro, Sunset Point; 9.3556°N, 82.2612°W; among *Thalassia*; 6 Aug 2005; R. Collin, M. Faust, E. Gaiser, S. LeCroy, S. Richardson, M. Sorensen leg.; GCRL 6694 • 1 ♂; Bocas del Toro, Hospital Point; 9.3336°N, 82.2188°W; depth 15 m; among coral rubble and *Halimeda*; 6 Aug 2005; S. DeGrave, M. Salazar leg.; GCRL 6695 • 11 ♂, 14 ♀, 6 juveniles; Bocas del Toro, Crawl Caye; 9.2505°N, 82.1316°W; depth 10 m; among coral rubble and red sponges; 7 Aug 2005; S. DeGrave, M. Salazar leg.; GCRL 6696 • 2 ♀; Bocas del Toro, Cayo Solarte; 9.3336°N, 82.2189°W; depth 2 m; among sand and coral rubble; 7 Aug 2005; R. Collin, M. Faust, E. Gaiser, S. LeCroy, S. Richardson, M. Sorensen leg.; GCRL 6697 • 1 ♂; Bocas del Toro, Weather Instrument Platform; 9.3333°N, 82.2185°W; depth 1 m; commensal in lavender sponge; 8 Aug 2005; T.A. Haney leg.; GCRL 6698 • 1 ♂; Bocas del Toro, Isla San Cristobal; depth 0.2 m; among red algae; 9 Aug 2005; T.A. Haney leg.; GCRL 6699 • 1 ♀; Bocas del Toro, Almirante; 9.2900°N, 82.3429°W; depth 10–11 m; commensal in *Pseudoceratina crassa*; 7 Aug 2021; K.N. White leg.; USNM 1762969 • 1 ♂; Bocas del Toro, Crawl Caye; 9.2378°N, 82.1438°W; depth 1.5–3 m; among coral rubble; 11 Aug 2021; K.N. White leg.; USNM 1762970 • 1 ♂; Bocas del Toro, San Cristobal; 9.2849°N, 82.2945°W; depth 1–3 m; among sponges; 21 June 2023; K.N. White leg.; USNM 1762971 • 5 ♀; Bocas del Toro, Hospital Point; 9.3319°N, 82.2148°W; depth 1–3 m; among coral rubble; 22 June 2023; K.N. White leg.; USNM 1762972 • 1 ♂; Bocas del Toro, Crawl Caye; 9.2459°N, 82.1369°W; depth 1–4 m; in sand; 25 June 2023; K.N. White leg.; USNM 1762973 • 2 juveniles; Bocas del Toro, STRI dock; 9.3511°N, 82.2570°W; depth 0–1 m; dock scraping; 27 June 2023; K.N. White leg.; USNM 1762971.

**Figure 20.**
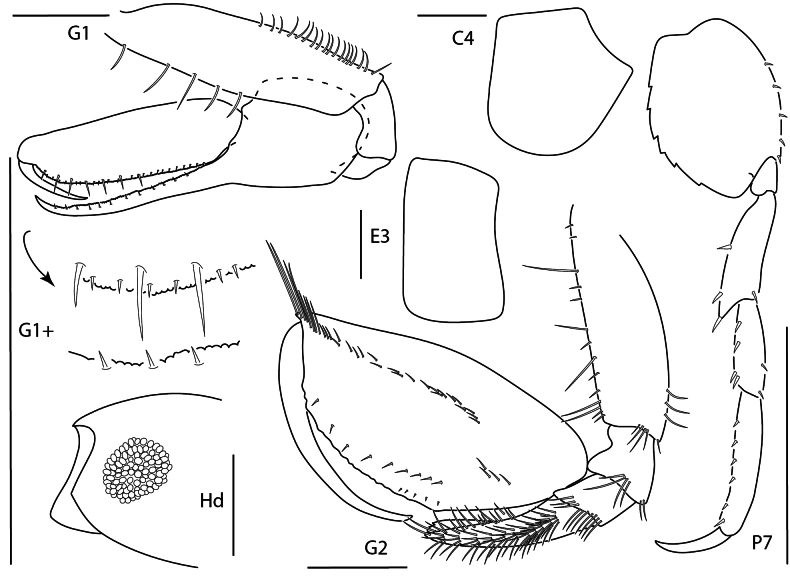
*Leucothoe ubouhu*, female, 3 mm, gnathopod 1 medial, epimeron 3, coxa 4, pereopod 7, head, gnathopod 2 medial. Scale bars: 0.5 mm.

##### Diagnosis.

Head anterior margin truncate, anterodistal margin with cusp; ventral keel produced, subquadrate. Gnathopod 1 basis anterior margin with several setae, female posterior margin lined with setae; carpus proximal margin dentate; propodus palm dentate, with distal setae; dactylus reaching further than 0.2 × propodus length. Gnathopod 2 basis anterior margin with several short and medium length setae, female posterior margin with few setae; carpus distally truncate, anterior margin dentate; propodus primary mediofacial setal row above midline, slightly displaced, reaching 0.6–0.7 × propodus length, secondary mediofacial setal row with four setae, palm minutely tuberculate, one row of submarginal setae present. Coxa 4 smooth, posterior margin concave. Epimeron 3 posteroventral corner subquadrate.

##### Distribution.

USA: Florida Keys (Thomas and Klebba 2007); Belize: Pelican Cays (Thomas and Klebba 2007); Panama: Bocas del Toro (White 2011; present study).

##### Ecology and remarks.

This species occurs as commensal in sponges, and among *Halimeda*, sand, and coral rubble at depths of 0–12 m. Panamanian specimens agree closely with the original description with the exception of a more setose posterior margin on gnathopod 1 basis (see White 2011 for other variations not found in these specimens). Living specimens are translucent orange with red eyes.

#### ﻿ Leucothoe wuriti

Taxon classificationAnimaliaAmphipodaLeucothoidae

Thomas & Klebba, 2007

3A471CEF-7C5A-5487-880D-A3D230421B56

Figs 21, 24F

Leucothoe spinicarpa : Ortiz 1975: 10, fig. 5.
Leucothoe wuriti Thomas & Klebba, 2007: 30–35, figs 19, 21, 22C, D; LeCroy 2011: 634–635, fig. 513; White 2011: 31–32, fig. 9.

##### Material examined.

Panama • 3.6–7 mm • 2 ♀; Bocas del Toro, Hospital Point; 9.3336°N, 82.2188°W; depth 15 m; among coral rubble and *Halimeda*; 6 Aug 2005; S. DeGrave, M. Salazar leg.; GCRL 6700 • 1 ♂, 1 ♀; Bocas del Toro, Hospital Bight; 9.3045°N, 82.1316°W; depth 1.5 m; among sponges, coral rubble and sand; 7 Aug 2005; R. Collin, M. Faust, E. Gaiser, S. LeCroy, S. Richardson, M. Sorensen leg.; GCRL 6701 • 1 ♀; Bocas del Toro, Laboratory Dock; 9.3511°N, 82.2570°W; depth 1 m; light trap; 8 Aug 2005; S. DeGrave leg.; GCRL 6702 • 2 juveniles; Bocas del Toro, Weather Instrument Platform; 9.3333°N, 82.2185°W; depth 1 m; commensal in lavender sponge; 8 Aug 2005; T.A. Haney leg.; GCRL 6703 • 1 ♂, 3 ♀; Bocas del Toro, Isla Solarte; 9.2901°N, 82.1897°W; depth 1–5 m; commensal in ascidian and mangrove scraping; 8 Aug 2021; K.N. White leg.; USNM 1762975.

**Figure 21.**
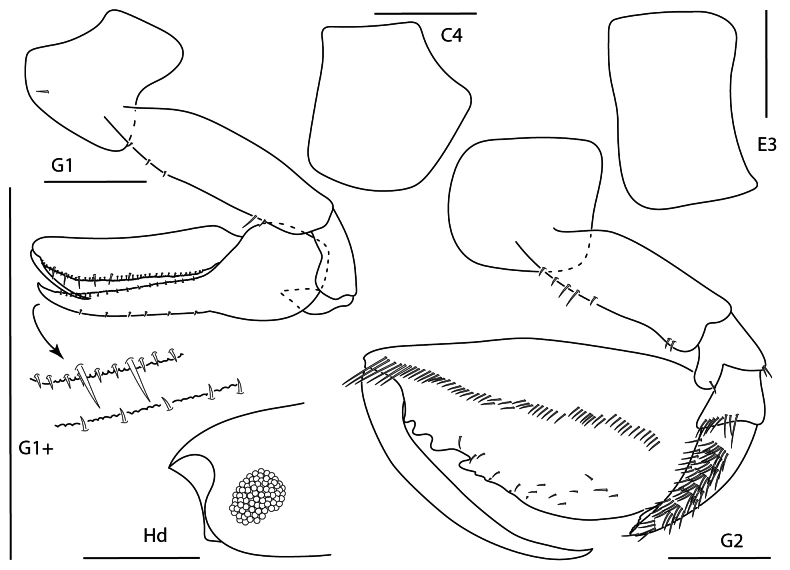
*Leucothoe wuriti*, male, 6.7 mm, gnathopod 1 medial, coxa 4, epimeron 3, head, gnathopod 2 medial. Scale bars: 0.5 mm.

**Figure 22.**
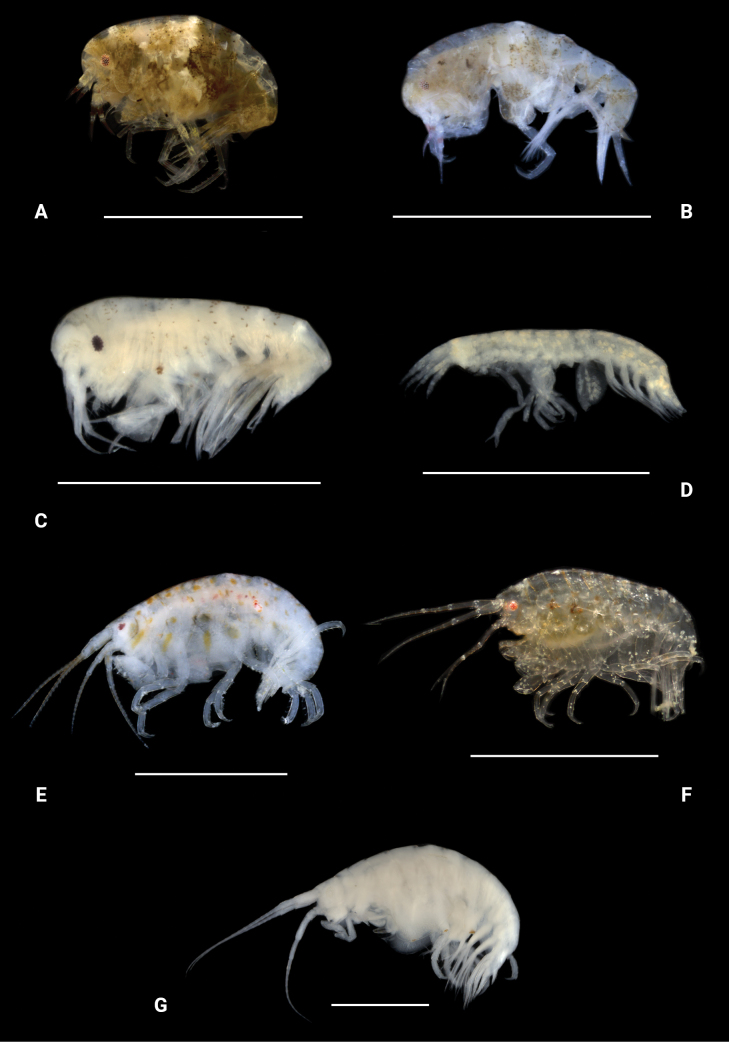
Photographs of living specimens unless noted. A. *Apolochus dragensis* sp. nov.; B. *Apolochus pillaii*; C. Apolochus cf. picadurus (ethanol-preserved specimen); D. Seba cf. tropica (ethanol-preserved specimen); E. *Stenothoe gallensis*; F. *Stenothoe minuta*; G. *Stenothoe valida* (ethanol-preserved specimen). Scale bars: 1.0 mm.

**Figure 23.**
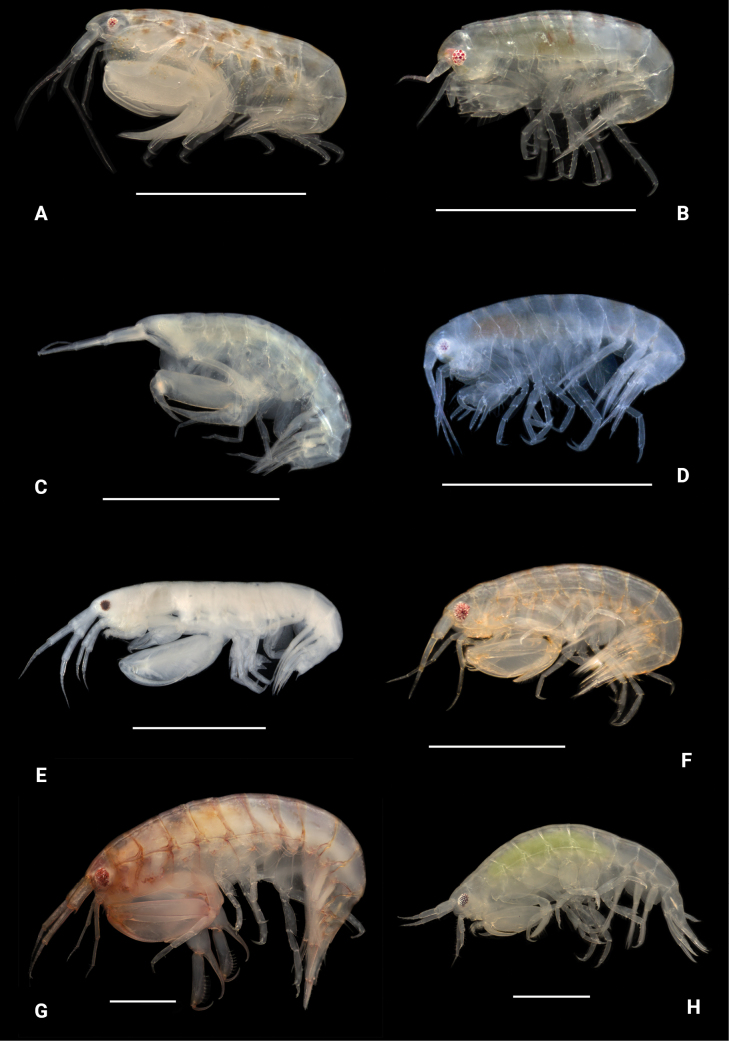
Photographs of live specimens unless noted. A. *Anamixis cavatura* anamorph; B. *Anamixis cavatura* leucomorph; C. *Anamixis vanga* anamorph (ethanol-preserved specimen); D. *Anamixis vanga* leucomorph; E. *Leucothoe alata* (ethanol-preserved specimen); F. *Leucothoe ashleyae*; G. *Leucothoe barana*; H. *Leucothoe flammosa*. Scale bars: 1.0 mm.

**Figure 24.**
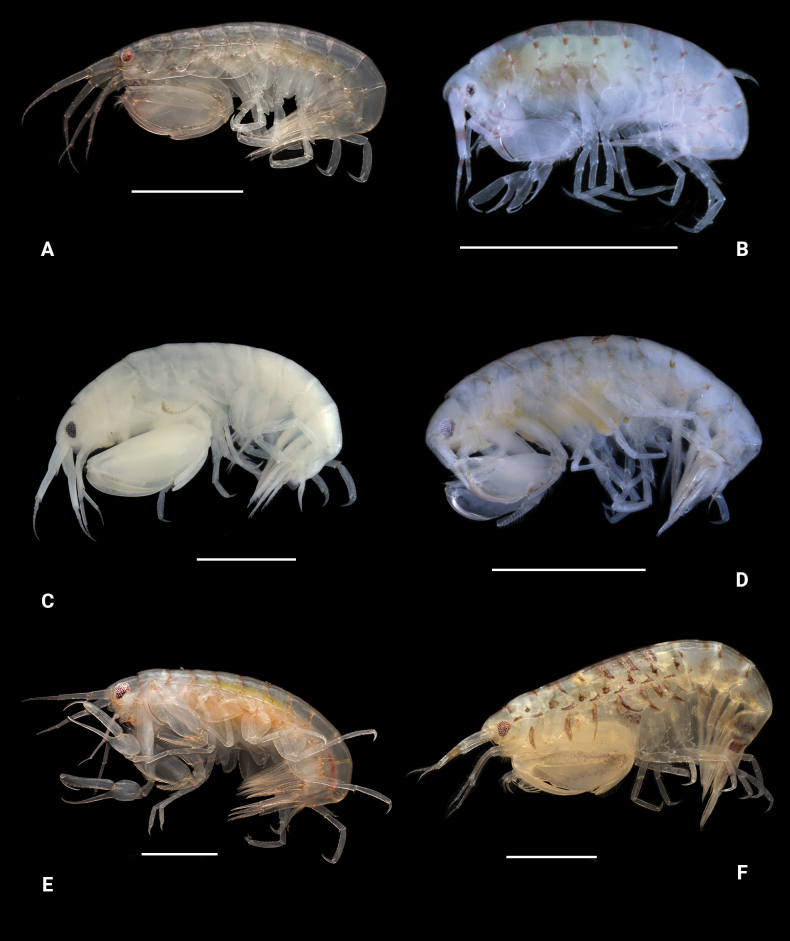
Photographs of living specimens unless noted. A. *Leucothoe kensleyi*; B. *Leucothoe laurensi*; C. *Leucothoe machidai* (ethanol-preserved specimen); D. *Leucothoe tunica* (ethanol-preserved specimen); E. *Leucothoe ubouhu*; F. *Leucothoe wuriti*. Scale bars: 1.0 mm.

##### Diagnosis.

Head anterior margin rounded; ventral keel anterodistal margin quadrate. Gnathopod 1 coxa with one mediofacial seta; basis anterior margin with few short setae, posterior margin bare; carpus proximal margin dentate, with several facial setae; propodus palm dentate; dactylus reaching more than 0.2× propodus length. Gnathopod 2 basis anterior margin with several short setae, posterior margin bare; carpus distally truncate, anterior margin dentate; propodus primary mediofacial setal row displaced to midline, reaching 0.8 × propodus length, secondary mediofacial setal row with three setae, palm with distinct tubercules, one row of submarginal setae present. Coxa 4 smooth, posterior margin concave. Epimeron 3 posteroventral corner subquadrate, slightly produced.

##### Distribution.

USA: Biscayne Bay, Florida Keys (Thomas and Klebba 2007); Belize: Pelican Cays (Thomas and Klebba 2007); Panama: Bocas del Toro (White 2011; present study).

##### Ecology and remarks.

This species occurs as commensal in ascidians and among sponges, coral rubble, *Halimeda*, and sand at depths of 0–15 m. Panamanian specimens agree closely with the original description except for a shorter facial seta on gnathopod 1 coxa (see White 2011 for other variations in these specimens). Living specimens are translucent white with maroon stripes on antenna 1 and along pereonite edges.

### ﻿Identification Key to the Caribbean Amphilochidira of Panama

**Table:** 

1	Eyes absent or poorly developed; gnathopods 1 and 2 chelate; urosome segments 2 and 3 fused (Fig. 6)	**Seba cf. tropica**
–	Eyes present, well-developed (Fig. 1); gnathopods 1 and 2 carpochelate (Fig. 11) or subchelate (Fig. 2); urosome segments 1–3 separate (Fig. 8)	**2**
2	Coxa 4 extensively broadened (Fig. 8); pereopod 5 basis not expanded (Fig. 9); uropod 3 uniramous (Fig. 8)	**3**
–	Coxa 4 not extensively broadened (Fig. 19); pereopod 5 basis expanded (Fig. 18); uropod 3 biramous (Fig. 5)	**5**
3	Gnathopod 2 propodus palmar angle present; uropod 1 inner ramus bare; uropod 3 second article of ramus 1.6× length of first article of ramus (Fig. 8)	***Stenothoe minuta***
–	Gnathopod 2 propodus palmar angle absent; uropod 1 inner ramus with at least one spine-seta; uropod 3 second article of ramus subequal in length with first article of ramus (Figs 7, 9)	**4**
4	Eye small; pereopod 7 merus posterodistal lobe not reaching more than halfway to distal margin of carpus; uropod 1 peduncle with distoventral spur (Fig. 7)	***Stenothoe gallensis***
–	Eye large; pereopod 7 merus posterodistal lobe expanded, nearly reaching distal margin of carpus; uropod 1 peduncle lacking distoventral spur (Fig. 9)	***Stenothoe valida***
5	Antenna 1 shorter than antenna 2 (Fig. 1); gnathopod 1 subchelate; propodus with single row of simple robust setae along entire palmar margin (Fig. 5)	**6**
–	Antenna 1 subequal in length with or longer than antenna 2; gnathopod 1 carpochelate; propodus lacking simple robust setae along entire palmar margin (slender setae may be present) (Fig. 21)	**8**
6	Head anteroventral angle acute; antenna 1 lacking accessory flagellum; gnathopod 2 propodus with small anterodistal projection (Fig. 4)	**Apolochus cf. picadurus**
–	Head anteroventral angle subquadrate or rounded; antenna 1 with minute uni-articulate accessory flagellum (Fig. 1); gnathopod 2 propodus lacking anterodistal projection (Fig. 2)	**7**
7	Head anteroventral angle rounded (Fig. 1); gnathopod 2 carpal lobe nearly reaching propodus palmar angle; propodus lacking antero-lateral spines, anterodistal margin rounded (Fig. 2)	***Apolochus dragensis* sp. nov.**
–	Head anteroventral angle subquadrate; gnathopod 2 carpal lobe not reaching propodus palmar angle; propodus with antero-lateral spines, anterodistal margin acute (Fig. 5)	***Apolochus pillaii***
8	Gnathopod 1 carpus and propodus each with long apical seta (Fig. 10G1a); gnathopod 2 ischium elongate, length at least 2× width (Fig. 10G2a)	**9**
–	Gnathopod 1 carpus and propodus lacking long apical setae (Fig. 10G1l); gnathopod 2 ischium not elongate, length < 2× width (Fig. 10G2l)	**10**
9	Head anterior margin rounded, anterodistal margin with cusp, without lateral ridge; ventral keel ventrally triangular; gnathopod 2 dactylus proximal margin with two large tubercles, distally serrate (Fig. 10)	***Anamixis cavatura* anamorph male**
–	Head anterior margin excavate, anterodistal margin without cusp, with lateral ridge; ventral keel ventrally quadrate; gnathopod 2 dactylus proximal margin with one large tubercle, distally smooth (Fig. 11)	***Anamixis vanga* anamorph male**
10	Gnathopod 1 carpus apex with spines (Fig. 10G1l); gnathopod 2 propodus subtriangular (Fig. 10G2l)	**11**
–	Gnathopod 1 carpus apex without spines; gnathopod 2 propodus subovate or subrectangular (Fig. 19)	**12**
11	Head anterior margin truncate; ventral keel projected upward; gnathopod 1 carpus proximal margin dentate, apex with two dentate embedded spines, larger spine distally bifid; gnathopod 2 propodus palm nearly transverse (Fig. 10l)	***Anamixis cavatura* leucomorph male or female**
–	Head anterior margin rounded; gnathopod 1 carpus proximal margin serrate, apex with two smooth embedded spines, larger spine distally expanded; gnathopod 2 propodus palm oblique (Fig. 11l)	***Anamixis vanga* leucomorph male or female**
12	Gnathopod 1 stout, propodus inflated; gnathopod 2 mediofacial setal row reaching almost entire length of propodus above midline (Figs 12, 17)	**13**
–	Gnathopod 1 linear, propodus straight; gnathopod 2 mediofacial setal row reaching 0.8× length of propodus or less, at least slightly displaced toward midline (Figs 13, 19)	**14**
13	Gnathopod 1 carpus with long distal seta, dactylus longer than 0.2× propodus length; gnathopod 2 propodus with large distal blade-like tooth, carpus reaching palmar margin, narrowing distally (Fig. 17)	***Leucothoe laurensi***
–	Gnathopod 1 carpus without long distal seta, dactylus shorter than 0.2× propodus length; gnathopod 2 propodus without large distal blade-like tooth, carpus not reaching palmar margin, distal margin spoon-like (Fig. 12)	***Leucothoe alata***
14	Gnathopod 1 basis proximally inflated, carpus with long distal setae; propodus palm smooth; dactylus reaching < 0.2× propodus length, nail-like; gnathopod 2 propodus with dense submarginal setae, palm defined by notch for insertion of dactylus; dactylus reaching ~0.5× propodus length (Fig. 15)	***Leucothoe flammosa***
–	Gnathopod 1 basis straight, carpus lacking long distal setae; propodus palm with ornamentation; dactylus reaching > 0.2× propodus length; gnathopod 2 propodus with sparse submarginal setae, palm not defined by notch for insertion of dactylus; dactylus reaching > 0.5× propodus length (Figs 19, 21)	**15**
15	Coxa 1 with mediofacial seta; gnathopod 2 propodus mediofacial setal row strongly displaced to midline, reaching 0.8× propodus length, palm with deep indentation between large projections (Fig. 21)	***Leucothoe wuriti***
–	Coxa 1 lacking mediofacial seta; gnathopod 2 propodus mediofacial setal row above midline, may be slightly displaced toward midline, reaching < 0.8× propodus length, palm without deep indentation (Fig. 13)	**16**
16	Head anterior margin evenly rounded; ventral keel anterior margin deeply concave, anteroventral margin rounded; gnathopod 2 carpus strongly expanded (Figs 13, 19)	**17**
–	Head anterior margin truncate; ventral keel anterior margin straight or weakly concave, anteroventral margin quadrate or subquadrate; gnathopod 2 carpus rounded, tapered, or slightly expanded (Figs 14, 16)	**18**
17	Gnathopod 1 propodus palm serrate; gnathopod 2 carpus distal margin oblique; coxa 4 posterior margin strongly concave (Fig. 13)	***Leucothoe ashleyae***
–	Gnathopod 1 propodus palm dentate; gnathopod 2 carpus distal margin rounded; coxa 4 posterior margin weakly concave/tapered (Fig. 19)	***Leucothoe tunica***
18	Head anteroventral margin with acute cusp; ventral keel with acute cusp; coxa 4 ventral margin tapered, serrate; pereopods 5–7 bases narrowly expanded, length > 1.4× width (Fig. 16)	**19**
–	Head anteroventral margin without pronounced cusp; ventral keel without pronounced cusp; coxa 4 ventral margin rounded, smooth; pereopods 5–7 bases widely expanded, length < 1.4× width (Fig. 20)	**20**
19	Ventral keel anterior margin oblique, projecting forward; gnathopod 1 carpus with three distolateral facial setae; coxa 4 anteroventral corner rounded; epimeron 3 posteroventral margin rounded (Fig. 14)	***Leucothoe barana***
–	Ventral keel anterior margin straight; gnathopod 1 carpus lacking distolateral facial setae; coxa 4 anteroventral corner acute; epimeron 3 posteroventral margin quadrate (Fig. 16)	***Leucothoe kensleyi***
20	Ventral keel anterior margin slightly convex, anteroventral margin serrate; female gnathopod 1 basis posterior margin bare; gnathopod 2 carpus distally rounded; coxa 4 posterior margin tapered (Fig. 18)	***Leucothoe machidai***
–	Ventral keel anterior margin straight, anteroventral margin smooth; female gnathopod 1 basis posterior margin with several long setae; gnathopod 2 carpus slightly expanded and oblique distally; coxa 4 posterior margin concave (Fig. 20)	***Leucothoe ubouhu***

## ﻿Discussion

This study documents a range extension for eight amphilochidiran amphipod species to include the Caribbean waters of Panama. Three species now have a distribution pattern including both the Pacific and Caribbean (Apolochus cf. picadurus, *Leucothoe alata*, and *Leucothoe kensleyi*). These distribution patterns may suggest that these species were established before the isthmus of Panama closed or may more likely be due to the clinging and commensal lifestyle of these species. These amphipods may be introduced to new areas with fouling organisms on ships traveling through the Panama Canal. *Apolochus dragensis* sp. nov. is described herein, increasing the known members of the genus to 16 species. This study increases the known number of amphilochidiran amphipods from Caribbean Panama from 12 to 21 species. The Caribbean Amphipoda of Panama identification key is available online: https://www.invertebase.org/portal/ident/key.php?clid=58&pid=4&dynclid=0&taxon=All+Species (Caribbean Amphipoda of Panama, 2025).

## Supplementary Material

XML Treatment for Apolochus

XML Treatment for Apolochus dragensis

XML Treatment for Apolochus cf. picadurus

XML Treatment for Apolochus pillaii

XML Treatment for Seba

XML Treatment for Seba cf. tropica

XML Treatment for Stenothoe

XML Treatment for Stenothoe gallensis

XML Treatment for Stenothoe minuta

XML Treatment for Stenothoe valida

XML Treatment for Anamixis

XML Treatment for Anamixis cavatura

XML Treatment for Anamixis vanga

XML Treatment for Leucothoe

XML Treatment for Leucothoe alata

XML Treatment for Leucothoe ashleyae

XML Treatment for Leucothoe barana

XML Treatment for Leucothoe flammosa

XML Treatment for Leucothoe kensleyi

XML Treatment for Leucothoe laurensi

XML Treatment for Leucothoe machidai

XML Treatment for Leucothoe tunica

XML Treatment for Leucothoe ubouhu

XML Treatment for Leucothoe wuriti

## ﻿Additional information

### Use of AI

No use of AI was reported.

### Author ORCIDs

Abigail R. Dugger https://orcid.org/0009-0007-9032-2052

Kristine N. White https://orcid.org/0000-0002-5203-1656

### Data availability

All of the data that support the findings of this study are available in the main text.
